# Supplementary material for: Multistep orthophosphate release tunes actomyosin energy transduction
Source: Nat Commun. 2022 Aug 5;13:4575. doi: 10.1038/s41467-022-32110-9 (PMC9356070; doi:10.1038/s41467-022-32110-9)
Supplement: Supplementary file 1 — Supplementary Information [file 41467_2022_32110_MOESM1_ESM.pdf]

Supplementary Material

Multistep orthophosphate release tunes actomyosin energy transduction

Simnon source code for statistical mechanokinetic models

%: Denotes “comment”

%: Note 1: Commands can be written in any order in Simnon except the declaration of the %Continuous system” and the “End” command

%Note 2: Symbols for states and parameters differ from those used in paper because 1. the %Simnon implementation has been developed from previous work and changes from the %previous terminology was not made to avoid errors and 2. some terminology used in the paper is %not valid Simnon syntax.

1. Simulations of results in Fig. 4c
continuous system PV7vinew %Declaration of continuous system (“program”)

%---
% Declaration (“state”) of state variables and their derivatives (“der”)
state a00 a0 a11 a111 a1 a2 a22 a3 i stif
state ia1 ia2 atpas Na str
der da00 da0 da11 da111 da1 da2 da22 da3
der di dstif dia1 dia2 datpas dNa dstr
%---

%Velocity of shortening (nm/s)
v:10000
%---
%Declaration of independent variable
time t

%Transformation of independent variable to distance x in nm
x=5-t

%---
%Definition of states
"a00:MT; a0:MDP; a1:AMDP
"a11: $AMDP_{PiR}/AMDP_{L}$ a111:AMD_{L} a2:$AMDP_{H}$; a22:AMD_{H} a3:AM/AMT/AMD
%---

%Differential equations
%--
%Functions defining derivatives of states
pa00=-(-kf*a00+kb*a0+koff*a3)/v
pa0=-(kf*a00+kp_m*a1-(kon+kb)*a0)/v
"Neglect weak-binding state
pa1=-(kon*a0+kp_min*a11-(kp_m+kp_plusr)*a1)/v
pa11=-(kminus*a2+kp_plusr*a1+kpp*(Pi/Kp)*a111-(kplus+kp_min+kpp)*a11)/v

```
pa111=-(kminus*a22+kpp*a11-(kpp*(Pi/Kp)+kplus)*a111)/v
pa2=-(kplus*a11+kminus2*a3+kpp*(Pi/Kp)*a22-(kminus+kpp+kd)*a2)/v
pa22=-(kplus*a111+kminus2*a3+kpp*a2-(kminus+kd+kpp*(Pi/Kp))*a22)/v
pa3=-(kd*(a2+a22)-(2*kminus2+koff)*a3)/v
```

%System of ordinary differential equations

```
da00=-pa00
da0=-pa0
da1=-pa1
da11=-pa11
da111=-pa111
da2=-pa2
da22=-pa22
da3=-pa3
%--------------------------------------------

%--------------------------------------------
```

%Cross-bridge stiffness at different x

```
ks01=if x>x1 then k else km01
ks1=if x>x11 then k else km1
ks2=if x>x2 then k else km2
ks3=if x>x3 then k else km3
%--------------------------------------------
%--------------------------------------------
```

"Note: l below is small L not "one"
% pia3: Function corresponding to force due to state a3 averaged
% over 36 nm. Includes possibility to have non-linear cross-bridge elasticity
%approximated by piecewise linear function

```
p4=kmh*(-4)
p70=kml*(-75-(-4))
pa3p=(1/36)*ks3*(x-x3)*a3
pa3h=(1/36)*a3*kmh*(x-x3)
pa3l=(1/36)*a3*(kml*(x-x3-(-4))+p4)
pa3ll=(1/36)*a3*(k*(x-x3-(-75))+p4+p70)
pa3l_est=if x-x3>(-75) then pa3l else if x-x3>(-90) then pa3ll else 0
pia3=if x>x3 then pa3p else if x-x3>-4 then pa3h else pa3l_est

fa314=kmh*(-4)+kml*(x-x3+4)
fa317=kml*(-75-(-4))
fa3147=kmh*(-4-x3)+fa317+k*(x-x3+75)
fa31=if x-x3>-75 then fa314 else if x-x3>90 then fa3147 else 0
fa3=if x>x3 then k*(x-x3) else if x-x3>-4 then kmh*(x-x3) else fa31

%--------------------------------------------
```

%Calculation of values for observable variables by integrating over all x and averaging

```
%------------------------------
```

% Stiffness due to all attached cross-bridges

```
dstif=(ks01/36)*(a1)+(1/36)*(ks1*(a11+a111)+ks2*(a2+a22))+(ks3/36)*a3
%------------------------------
```

%Fraction of attached cross-bridges

```
dNa=(a1+a11+a111+a2+a22+a3)/36
%------------------------------
```

% Force:

```
pis=(1/36)*(36*pia2+36*pia1+ks01*a1*(x-x1))+pia3
pistr=(1/36)*((a2+a22)*(x-x2)+(a11+a111)*(x-x11)+a3*(x-x3))
pia2=(ks2/36)*(a2+a22)*(x-x2)
pia1=(ks1/36)*(a11+a111)*(x-x11)
di=pis
dia1=pia1
dia2=pia2
dia3=pia3
dstr=pistr
%------------------------------
```

% ATPase

```
datpas=(1/36)*koff*a3
%--------------------------------------------

%--------------------------------------------
```

%Assignment of initial values

```
a00:0.1
a0:0.9
a1:0
a11:0
a111:0
a2:0
a22:0
a3:0
%--------------------------------------------

%--------------------------------------------
```

%Rate functions for attachment kon$_+$(x) (kon) and reversal
% of attachment kon$_-$(x) (kp_m).
%Divide k*x by 4 to transform to units of k_BT

```
kpi_plus:130
exp_s0=(ks01/2)*(x-x1)*(x-x1)/4-(0/2)*(x-x1)*(x-x1)/4
kon_l00=kpi_plus*exp(aktens0-exp_s0/2)
kof_l00=kpi_plus*exp(exp_s0/2)
kon_a=if kon_l00<0.0001 then 0 else kon_l00
kon=if abs(x-x1)<5 then kon_a else 0
kp_m_a=if kof_l00>fc then fc else kof_l00
kp_m=if abs(x-x1)<5 then kp_m_a else fc
%--------------------------------------------

%--------------------------------------------
```

%Rate functions for transitions between AMDP$_{PP}$ and AMDP$_L$ states .kp+(x) (kp_plusr) and kp-(x)
% (kp_min)

```
kp_plus:3000
```

e_ps0=akten1/2-(ks1/16)*(x-x11)*(x-x11)+(ks01/16)*(x-x1)*(x-x1)
kp_min0=kp_plus*exp(-e_ps0)
e1a=e_ps0
e1ma=ln(fc/kp_plus)
e1mi=ln(1e-6/kp_plus)
kp_plusr=if e1a<e1mi then 1e-6 else if e1a>e1ma then fc else kp_plus*exp(e_ps0)
kp_min=if kp_min0>fc then fc else kp_min0
%---

%---
%Rate functions for Huxley-Simmons transition; AMD$_L$-AMD$_H$ and AMDP$_L$-AMDP$_H$)
% i.e. klh+(x) (kplus) and khl-(x) (kminus)
kminus11:6000
exp_12=(ks1/2)*(x-x11)*(x-x11)/4-(ks2/2)*(x-x2)*(x-x2)/4
kplusf=kminus11*exp(akten2+exp_12)
kvot1=kminus11/kplusf
kplus=if (kplusf>fc1) then fc1 else kplusf **%klh+(x)**
kminus=kminus11 %**khl-(x)**
Keq12=if kplus/kminus>fc1 then fc1 else kplus/kminus **%KLH**
%---

%---
%k5(x) (kplusf2)
kmi2=kmin5
exp_2=(ks2/2)*(x-x2)*(x-x2)/4-Gam
Gam=if x>x3 then Gamp else if x>(-4-x3) then Gamh else Gaml
Gamp=(k/2)*(x-x3)*(x-x3)/4
Gamh=(kmh/2)*(x-x3)*(x-x3)/4
Gaml0=kmh*(-4)*(x-x3+4)/4
Gaml=(kml/2)*(x-x3+4)*(x-x3+4)/4+(kmh/2)*16/4+Gaml0
e23a=akten3+exp_2
e23ma=ln(fc/kmi2)
e23mi=ln(1e-6/kmi2)
kplusff2=if e23a<e23mi then 1e-6 else if e23a>e23ma then fc else kmi2*exp(e23a)
kplusf2=cf*kplusff2
kvot2=if kplusff2>0.00001 then kmi2/kplusff2 else kmi2/0.00001
cf:0
"Irreversibility (cf==0) is motivated by very low [MgADP]
kd=if (kplusff2>fc) then fc else kplusff2 **%k5(x)**
kminus2=if (kplusf2>(fc-1)) then kvot2*kplusff2 else kmi2
%---

%---
%koff(x)
"Parameters for Bell-detachment rates below
fb=exp(ks3*abs(x-x3)*xb/4)

koff0=fb*k2a*ATP/((1/(K1a))+ATP+(k2a*fb/k4)*ATP)
koff=if x<(x1+4*xlimit) then koff0 else 0
koftot=koff*kd/(koff+kd)
%--

%--
%Parameter values below can be found in Supplementary Tables 5, 6 and 7 under other names (as indicated in %comments)
kf:200 %k_{+3}
kb:20 %k_{-3}

kpp:100 %k_{P+}
Pi:0.5 %[Pi]
Kp:10 %K_C
kmin5:2000
k2a:2000 %k_2; ATP induced detachment rate
K1a:1.7 %K_1; ATP binding constant
ATP:5 %[ATP]
k4=k4a
k4a:5000 %k_6; ADP dissociation rate constant
xb:0.6 %x_{crit}: Bell strain dependence parameter

fc:100000 %Value of rate functions if certain maximum limit reached –for stable computations. Is % varied with velocity
fc1:100000 %Value of rate functions if certain maximum limit reached –for stable computations. Is % varied with velocity
%--

%--
%Free energy differences between states
aktens0:0.7 % $\Delta G_{AMDP\text{-}AMDPL}$
akten1:1 % ΔG_{on}
akten2:14 % $\Delta G_{AMDL\text{-}AMDH} = \Delta G_{AMDP'L\text{-}AMDP'H}$
akten3:2 % $\Delta G_{AMDH\text{-}AMD} = \Delta G_{AMDPH\text{-}AMD}$
%--

%Limitation of cross-bridge attachment rate in some rate functions above
xlimit:3
%--

%--
%Assigning positions of minima for free energies for different states
x1:7.2
x11:6.7
x2:1
x3:0
%--

%--
%Cross-bridge stiffness for different x-values
k:2.8
%Below, possibility of non-linear cross-bridge elasticity only in the AM/AMD state.
km10:0.30769
km12:0.03255
ck:1
"ck varies from 0 (k & Stiffness according to [1]) to 1 (linear)
kmh=km10+ck*(k-km10)
kml=km12+ck*(k-km12)
km3=if x>x3-4 then kmh else if x>x3-75 then kml else if x>x3-90 then k else 0
"km3=if x>x3-4 then kmh else if x>x3-75 then kml else kml
km2:2.8
km1:2.8
km01:2.8
kw:0.0001
%--
end

2. Simulation of results in Fig. 4d

continuous system PV7vnewt
%only linear cross-bridge elasticity assumed

%--
% Declaration ("state") of state variables and their derivatives ("der")
state a00 a0 a11 a1 a111 a2 a22 a3
state i stif ia1 ia2 atpas Na
der da00 da0 da11 da1 da111 da2 da22 da3
der di dstif dia1 dia2 datpas dNa
%--

%--
%Declaration of independent variable
time t
%--

%--
%Definition of states
"a00:MT; a0:MDP; a1:AMDP
"a11: $AMDP_{PiR}$/$AMDP_{L}$ a111:AMD_{L} a2:$AMDP_{H}$; a22:AMD_{H} a3:AM/AMT/AMD
%--

%Differential equations
%--

```
%Functions defining derivatives of states
pa00=-(-kf*a00+kb*a0+koff*a3)
pa0=-(kf*a00+kp_m*a1-(kon+kb)*a0)
"Neglect weak-binding state
pa1=-(kon*a0+kp_min*a11-(kp_m+kp_plusr)*a1)
ad111=kpp*(Pi/Kp)*a111
pa11=-(kminus*a2+kp_plusr*a1+ad111-(kplus+kp_min+kpp)*a11)
pa111=-(kminus*a22+kpp*a11-(kpp*(Pi/Kp)+kplus)*a111)
pa2=-(kplus*a11+kpp*(Pi/Kp)*a22-(kminus+kpp+kd)*a2)
pa22=-(kplus*a111+kpp*a2-(kminus+kd+kpp*(Pi/Kp))*a22)
pa3=-(kd*(a2+a22)-koff*a3)

%Differential equations
da00=-pa00
da0=-pa0
da1=-pa1
da11=-pa11
da111=-pa111
da2=-pa2
da22=-pa22
da3=-pa3
%--------------------------------------------


%--------------------------------------------
%Calculation of average cross-bridge displacement, l
den_l=(a1+a11+a111+a2+a22+a3)
l=((a11+a111)*h0+a2*(h0+h)+a22*(h0+h)+a3*(h0+h+h1))/den_l
%--------------------------------------------

%--------------------------------------------
%Assignment of cross-bridge stiffness
kx=k
%--------------------------------------------

%--------------------------------------------
%Integration to obtain average stiffness (stif), number of attached cross-bridges (Na)
%force (i) and ATPase (atapas)
dstif=(k/36)*(a1+a11+a111+a2+a22+a3)
dNa=a1+a11+a111+a2+a22+a3
pis=pia1+pia2+pia3
pia2=F_set*(a2+a22)
pia3=F_set*a3
pia1=F_set*(a111+a11+a1)
datpas=(1/36)*koff*a3
di=pis
dia1=pia1
```

```
dia2=pia2
%-------------------------------------------

%-------------------------------------------
%Calculation of change in x for given Force set-point, F_set
x=F_set/k
%-------------------------------------------

%-------------------------------------------
%Differences along x-axis of equal force in different states
h0=x1-x11
h=x11-x2
h1=x2-x3
%-------------------------------------------

%-------------------------------------------
%Assignment of force set point and initial values of different states
F_set:0
a00:0
a0:0
a1:1
a11:0
a111:0
a2:0
a22:0
a3:0
%-------------------------------------------

%-------------------------------------------
%Rate functions for attachment kon+(x) (kon) and reversal
% of attachment kon-(x) (kpi_plum).  Because we are not interested
% in new attachment events (starting with all cross-bridges in state
% a1; AMDP) but only power-stroke events, we set kon+(x)=0
%Divide k*x by 4 to transform to units of kBT
kpi_plus:0
kpi_plum:130
exp_s0=(1/2)*F_set*F_set/(k*4)
kon_l00=kpi_plus*exp(aktens0-exp_s0/2)
kof_l00=kpi_plum*exp(exp_s0/2)
kon=if kon_l00<0.0001 then 0 else kon_l00
kp_m=if kof_l00>fc then fc else kof_l00

%-------------------------------------------
%Rate functions for transitions kp+(x) (kp_plusr) and kp-(x) (kp_min)
kp_plus:3000
exp_ps0=akten1/2+(1/2)*F_set*F_set/(k*4)-(k/2)*(x+h0)*(x+h0)/4
"forward
exp_ps0m=akten1/2+(k/2)*(x-h0)*(x-h0)/4-(1/2)*F_set*F_set/(k*4)
```

kp_plur0=kp_plus*exp(exp_ps0)
kp_min0=kp_plus*exp(-exp_ps0m)
kp_plusr=if kp_plur0<0.0001 then 0 else kp_plur0
kp_min=if kp_min0>fc then fc else kp_min0
%--

%--
%Rate functions for Huxley-Simmons transition; AMD_L-AMD_H and $AMDP_L$-$AMDP_H$)
% i.e. klh+(x) (kplus) and khl-(x) (kminus)
kminus11:6000
exp_12=(1/2)*F_set*F_set/(k*4)-(k/2)*(x+h+h0)*(x+h+h0)/4
"forward
exp_12m=(k/2)*(x-(h+h0))*(x-(h+h0))/4-(1/2)*F_set*F_set/(k*4)
"backward
kplusf=kminus11*exp(akten2+exp_12)
"kpfh=if (akten2+exp_12m)>ln(fc1) then fc1 else kminus11*exp(akten2+exp_12m)
kpfh=kminus11*exp(akten2+exp_12m)
kvot1=kpfh/kminus11
kplus=if (kplusf>fc1) then fc1 else kplusf
Keq12=if kvot1>fc1 then fc1 else if kvot1<0.00001 then 0.00001 else kvot1
kminus=kminus11/Keq12
%--

Pi:0.5
Kp:10
"Dissociationconstant of Pi

%--
%k5(x) (kd)
kmin2=kmin5
xh=h1+F_set/k
xhh1=(x-h-h1-x3)
exp_2=(1/2)*F_set*F_set/(4*k)-(kx/2)*xh*xh/4
kplusf2=if kmin2*exp(akten3+exp_2)>fc1 then fc1 else kmin2*exp(akten3+exp_2)
kd=if (kplusf2>fc) then fc else kplusf2
%--

%Parameters for Bell-detachment rates below
%--
fb=exp(abs(F_set)*xb/4)
koff0=fb*k2a*ATP/((1/(K1a))+ATP+(k2a*fb/k4)*ATP)
koff=if x<(x1+4*xlimit) then koff0 else 0
koftot=koff*kd/(koff+kd)
%--

%---

%Parameter values below can be found in Supplementary Tables 5 and 6 under other names (as indicated in %comments)

kf:200 %k_{+3}
kb:20 %k_{-3}

kpp:100 %k_{P+}
Pi:0.5 %[Pi]
Kp:10 %K_C
kmin5:2000
k2a:2000 %k_2; ATP induced detachment rate
K1a:1.7 %K_1; ATP binding constant
ATP: 0.0001 %[ATP]
k4=k4a
k4a:5000 %k_6; ADP dissociation rate constant
xb:0.6 %x_{crit}: Bell strain dependence parameter

fc:100000 %Value of rate functions if certain maximum limit reached –for stable computations. Is % varied with velocity
fc1:100000 %Value of rate functions if certain maximum limit reached –for stable computations. Is % varied with velocity
%--

%--

%Free energy differences between states

aktens0:0.7 % $\Delta G_{AMDP-AMDPL}$
akten1:1 % ΔG_{on}
akten2:14 % $\Delta G_{AMDL-AMDH} = \Delta G_{AMDP'L-AMDP'H}$
akten3:2 % $\Delta G_{AMDH-AMD} = \Delta G_{AMDPH-AMD}$
%--

%Limitation of cross-bridge attachment rate in some rate functions above

xlimit:3
%--

%--

%Assigning positions of minima for free energies for different states

x1:7.2
x11:6.7
x2:1
x3:0
%--

%--

%Cross-bridge stiffness

k:2.8
km:2.8
%--

end
%---
%---

3. Simulation of results in Fig. 5

continuous system Pirel (Model in Supplementary Figure 8a)

%--
% Declaration ("state") of state variables and their derivatives ("der")
state a0 a1 a2 a11 a22 a3
der da0 da1 da2 da11 da22 da3
%--

%--
%Declaration of independent variable
time t
%--

%Differential equations
%---
da0=k10*a1+kon*a3-(kon_m+k01)*a0
da1=k01*a0+k21*a2*Pi+kmin*a11-(k10+k12+kplu)*a1
da2=k12*a1+kmin*a22-(k21*Pi+kplu)*a2
da11=kplu*a1+k2211*a22*Pi-(k1122+kmin+kdet)*a11
da22=kplu*a2+k1122*a11-(kmin+k2211*Pi+kdet)*a22
da3=kdet*(a11+a22)+kon_m*a0-kon*a3
%---

%Parameter values below can be found in Supplementary Figure 8 under indicated in %comments
%---
ks:2.5 %cross-bridge stiffness
kon:261 %k_{on}
kon_m:131 %k_{on-}
Pi:0.5 %[Pi], mM
k01:2000 %k_{Pr+}
k10:2000 %k_{Pr-}
k12:100 %k_{P+}
k21:10 %k_{P-}= k_{P+}/K_C ($s^{-1}mM^{-1}$)
k1122:100 %k_{P+} (other variable name than for k21 in program gives increased flexibility)
k2211:10 %k_{P-}= k_{P+}/K_C ($s^{-1}mM^{-1}$)
kplu:5000 %k_{LH+}
kmin:2500 %k_{LH-}
kdet:5 %
atp:5 %[MgATP], mM

%Initial values

```
%-----------------------------
a0:0
a1:0
a2:0
a11:0
a22:0
a3:1
%-----------------------------

%Calculation of force and number of attached cross-bridges (att)
%-----------------------------
force=ks*(a11+a22)
att=a0+a1+a2+a11+a22
%-----------------------------
End
%--------------------------------------------
%--------------------------------------------


continuous system Pirel3  (Model in Supplementary Figure 8b)
%--------------------------------------------
% Declaration ("state") of state variables and their derivatives ("der")
state a0 a1 a2 a11 a22 a3 a1b a11b
der da0 da1 da2 da11 da22 da3 da1b da11b
%--------------------------------------------

%--------------------------------------------
%Declaration of independent variable
time t
%--------------------------------------------

%Differential equations
%--------------------------------------------
da0=k10*a1+kon*a3-(kon_m+k01)*a0
da1=k01*a0+k1bm*a1b+kmin*a11-(k10+k1b+kplu)*a1
da1b=k1b*a1+kmin*a11b+k21*Pi*a2-(k12+k1bm+kplu)*a1b
da2=k12*a1b+kmin*a22-(k21*Pi+kplu)*a2
da11=kplu*a1+k11bm*a11b-(k11b+kmin+kdet)*a11
da11b=kplu*a1b+k11b*a11+k2211*a22*Pi-(k11bm+kdet+k1122+kmin)*a11b
da22=kplu*a2+k1122*a11b-(kmin+k2211*Pi+kdet)*a22
da3=kdet*(a11+a11b+a22)+kon_m*a0-kon*a3
%--------------------------------------------

%Parameter values below can be found in Supplementary Figure 9 under names indicated in
%comments
%--------------------------------------------
ks:2.5  %cross-bridge stiffness
kon:261       %kon
```

kon_m:131 %k_{on-}
Pi:0.5 %[Pi], mM
k01:2000 %k_{Pr+}
k10:2000 %k_{Pr-}
k12:20000 %k_{P+}''
k21:1000 %k_{P-}''= k_{P+}'' /K_C'' ($s^{-1}mM^{-1}$)
k1122:20000 %k_{P+}'' (other variable name than for k21 in program gives increased flexibility)
k2211:1000 %k_{P-}''= k_{P+}'' /K_C'' ($s^{-1}mM^{-1}$)
k1b:100 %k_{P+}
k11b:100 %k_{P+}
k1bm:350 %k_{P-}= k_{P+} /K_C (s^{-1})
k11bm:350 %k_{P-}== k_{P+}/K_C(s^{-1})
"mM^-1 s^-1
kplu:5000 %k_{LH+}
kmin:2500 %k_{LH-}

k01:2000 %k_{Pr+}
k10:2000 %k_{Pr-}
k12:100 %k_{P+}
k21:10 %k_{P-}= k_{P+}/K_C ($s^{-1}mM^{-1}$)
kdet:5

%Initial values
%----------------------------
a0:0
a1:0
a1b:0
a11b:0
a2:0
a11:0
a22:0
a3:1
%----------------------------

%Calculation of force and number of attached cross-bridges (att)
%----------------------------
force=ks*(a11+a11b+a22)
att=a0+a1+a2+a11+a22+a1b+a11b
tot=a0+a1+a2+a11+a22+a3+a1b+a11b
%----------------------------

End
%---
%---

Supplementary Figures

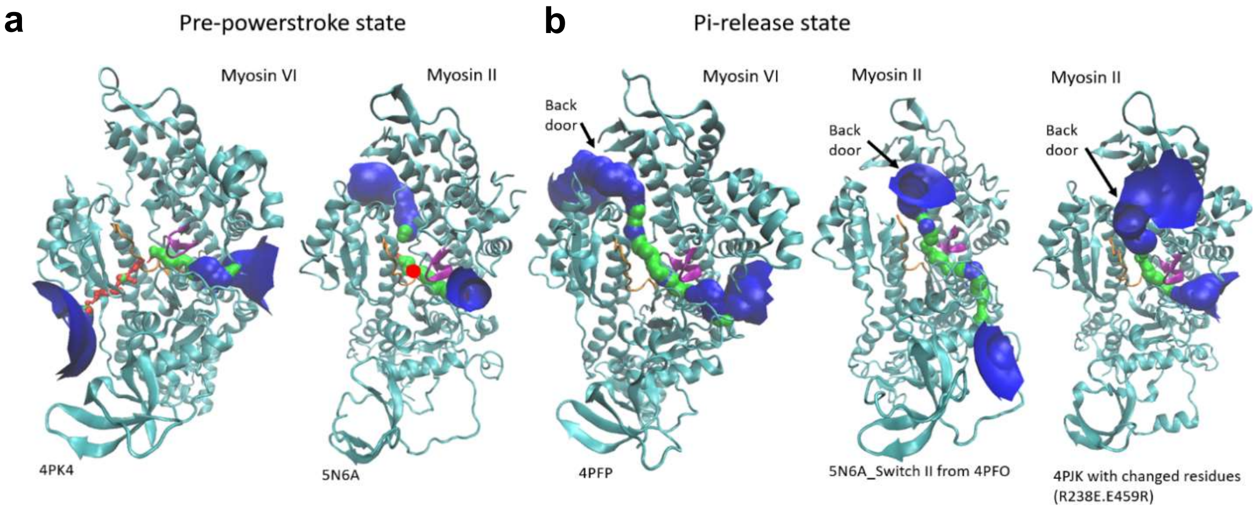

Supplementary Figure 1. Release tunnel in Pre-powerstroke (PPS) and Pi-release state (PiR). **a** Pre-power-stroke state. No fully open Pi-release tunnel is found (red mesh/red dot). This is the case both for myosin VI (PDB: 4PK4) and II (PDB: 5N6A). **b** The Pi-release state. A Pi-release tunnel, the 'back door', is open (blue and green mesh) for myosin VI (PDB: 4PFP), cardiac myosin II (5N6A_Switch II from 4PFO) and Dictyostelium myosin II (PDB: 4PJK; modelled after reversing mutations from the original study[2]). Specifically for the modelling of Dictyostelium myosin II with the two mutations R238E and E459R, as in the original crystal[2], no back door was found. Only, after exchanging the mutated amino acids for the originals using PyMol (version 1.8.6.2,) the back door was found as indicated. The program HOLE[3,4] was used to calculate the presence of the exit tunnel for the Pi, starting from ATP binding. Switch I: purple; Switch II: orange. Figures were made using VMD[5].

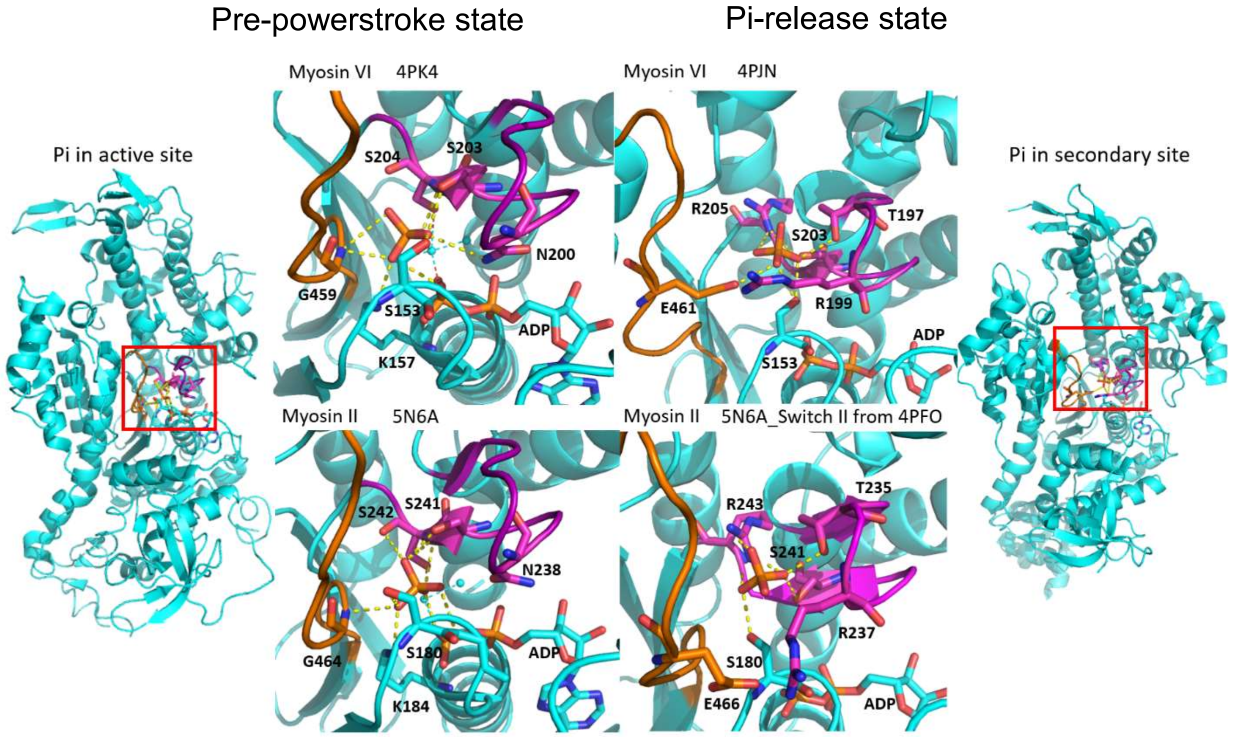

Supplementary Figure 2. Structural models of cardiac ventricular myosin II and myosin VI with a phosphate at the active site in the pre-powerstroke state (left) and in the secondary site in the Pi-release state (right). Models that were used for Pi in the active site in the pre-powerstroke states are based on PDB structures 5N6A (myosin II) and 4PK4 (myosin VI). Models for Pi in the secondary site in the Pi-release states are based on PDB structure 5N6A (myosin II) supplemented with the switch II conformation from PDB 4PFO (myosin VI). To allow release of the phosphate, it is necessary that Switch II moves from its initial pre-powerstroke state to the modelled state shown in orange. Hydrogen bonds indicated by yellow dashed lines. Figures were made using PyMol (The PyMol Molecular Graphics System, Version 2.0, Schrödinger, LLC). The panels showing data for myosin II are taken as basis for the image in Fig. 1c in the main paper.

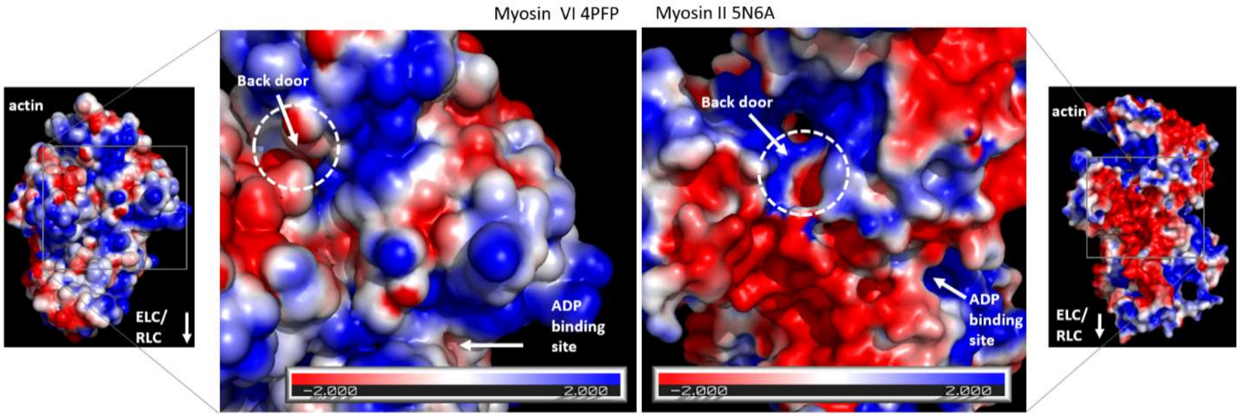

Supplementary Figure 3. Electrostatics around exit of back door for myosin II and myosin VI. Molecular models showing the electrostatic surface around opening of the back door in myosin II (PDB: 5N6A) and myosin VI (PDB: 4PFP). The electrostatic potential is colored from -2 k_BT (deep red) to +2 k_BT (deep blue). The figure was made using PyMol (The PyMol Molecular Graphics System, Version 2.0, Schrödinger, LLC) with the APBS extension[6].

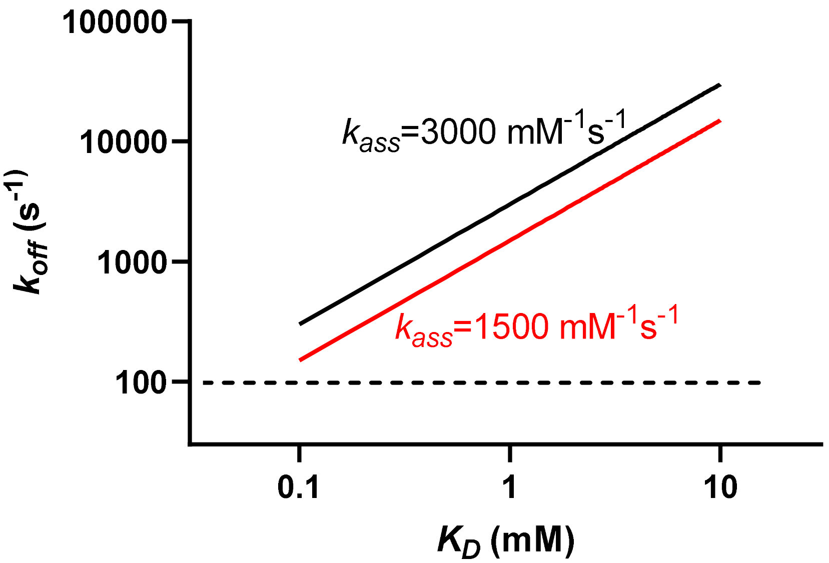

Supplementary Figure 4. Estimated values for the rate constant of dissociation (*k_{off}*) of Pi from tertiary weak binding site on surface of myosin head vs dissociation constant, *K_D*, in possible range according to main Fig. 2. By assuming a Michaelis-Menten constant K_M for the myosin ATPase in the range 50-100 nM and k_{cat}=0.05 s^{-1} ([7] and references therein) one can estimate the association rate constant of ATP as 0.5-1 x 10^6 M^{-1}s^{-1}. Now assuming that the latter rate constant is 3 times higher for inorganic phosphate (due to faster diffusion) and that this rate constant is similar for binding to the tertiary sites. Then, the association rate constant k_{ass} for Pi-binding to these sites would be in the range 1.5-3 x 10^6 M^{-1}s^{-1}. On this basis k_{off} is then calculated as k_{off}=K_D k_{ass}. Note that the calculated k_{off} values, for all values of K_D and k_{ass}, are higher than the range of rate constant of Pi-release from actomyosin found in transient kinetics studies[8,9]. Further, note that the estimates of k_{ass}, and thereby of k_{off} are most likely lower limits of the possible range as a pure diffusion limited rate constant of association is expected to be several orders of magnitude higher that the value we use here (cf. [10]).

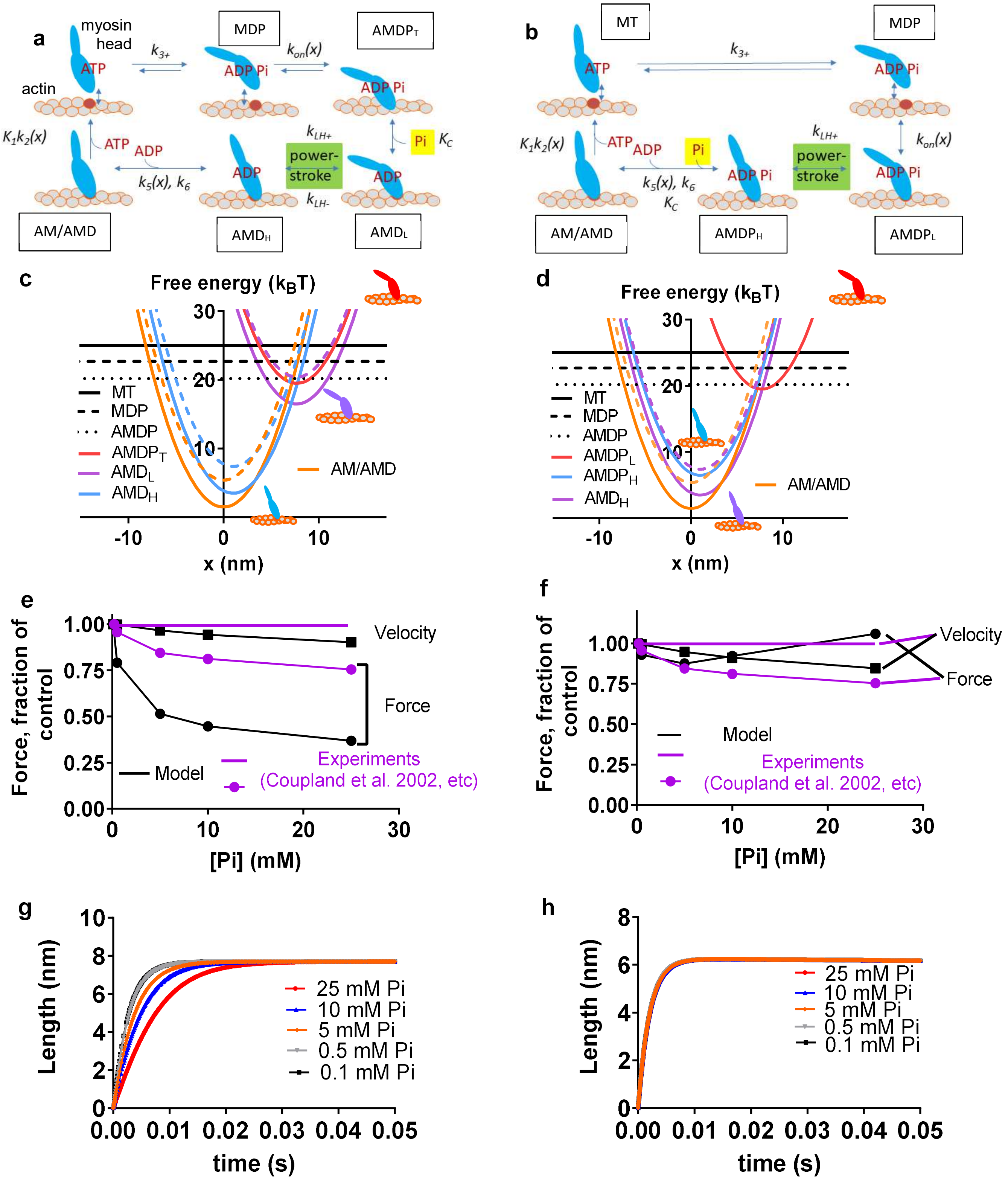

Supplementary Figure 5. Simplest models with Pi-release before and after power-stroke. a Cartoon illustrating model with Pi-release before the power-stroke based on [11-13]. **b** Cartoon

illustrating kinetic scheme for model modified from that in a to have Pi-release after the power-stroke. **c** Free energies of different cross-bridge states for model in a at 0.5 mM Pi (full coloured lines) and 25 mM Pi (dashed coloured lines). Black straight lines that refer to detached cross-bridges are not altered by changed [Pi]. Coarse-grain cross-bridge structures depicted for states $AMDP_T$, AMD_L and AMD_H as defined by color coding. **d** Free energies of model in d showing different cross-bridge states at 0.5 mM Pi and 25 mM Pi as indicated by colour coding. Coarse-grain cross-bridge structures depicted for states $AMDP_L$, $AMDP_H$ and AMD_H as defined by color coding. **e** Simulated steady-state values for maximum isometric force (black circles) and maximum velocity of shortening (black squares) using the model displayed in a and c, compared to experimental data for velocity (e.g. [14,15]) and force [16] at 30 °C. **f** Data plotted as in e but for the model in b and d. Note that whereas it may, at a first glance, appear that correspondence between model and experimental data is better than in e, the trends are wrong. Thus, unlike the experimental data the model predicts a monotonous decrease in velocity and a small decrease followed by a substantial increase in force with increased [Pi]. **g** Simulation, using the model displayed in a and c of power-strokes (nm displacement vs time) for an ensemble of myosin heads attaching in the pre-power stroke, actomyosin state with ADP and Pi at the active site and cross-bridge force clamped to 0 pN. Note, a strong dependence on [Pi] is predicted in contradiction with experimental results[17]. **h** Data as in g but for the model in b and d**.** Note, in agreement with experimental results[17], no dependence on [Pi] is predicted. Note, further, slightly different total power-stroke amplitudes for models in A and B. Model parameter values are for 30 °C and fast skeletal muscle myosin close to physiological ionic strength (Supplementary Tables 3 - 4). M: myosin; A: actin; T: ATP; D: ADP; P/Pi: inorganic phosphate; the subscripts "T", "L" and "H" denote "transient", low-force and high-force state, respectively. The free energies at 25 mM Pi are shifted upwards in both c and d to make the free energies of the MTP, MDP and the AMDP state (black lines) coincide at 0.5 mM and 25 mM Pi.

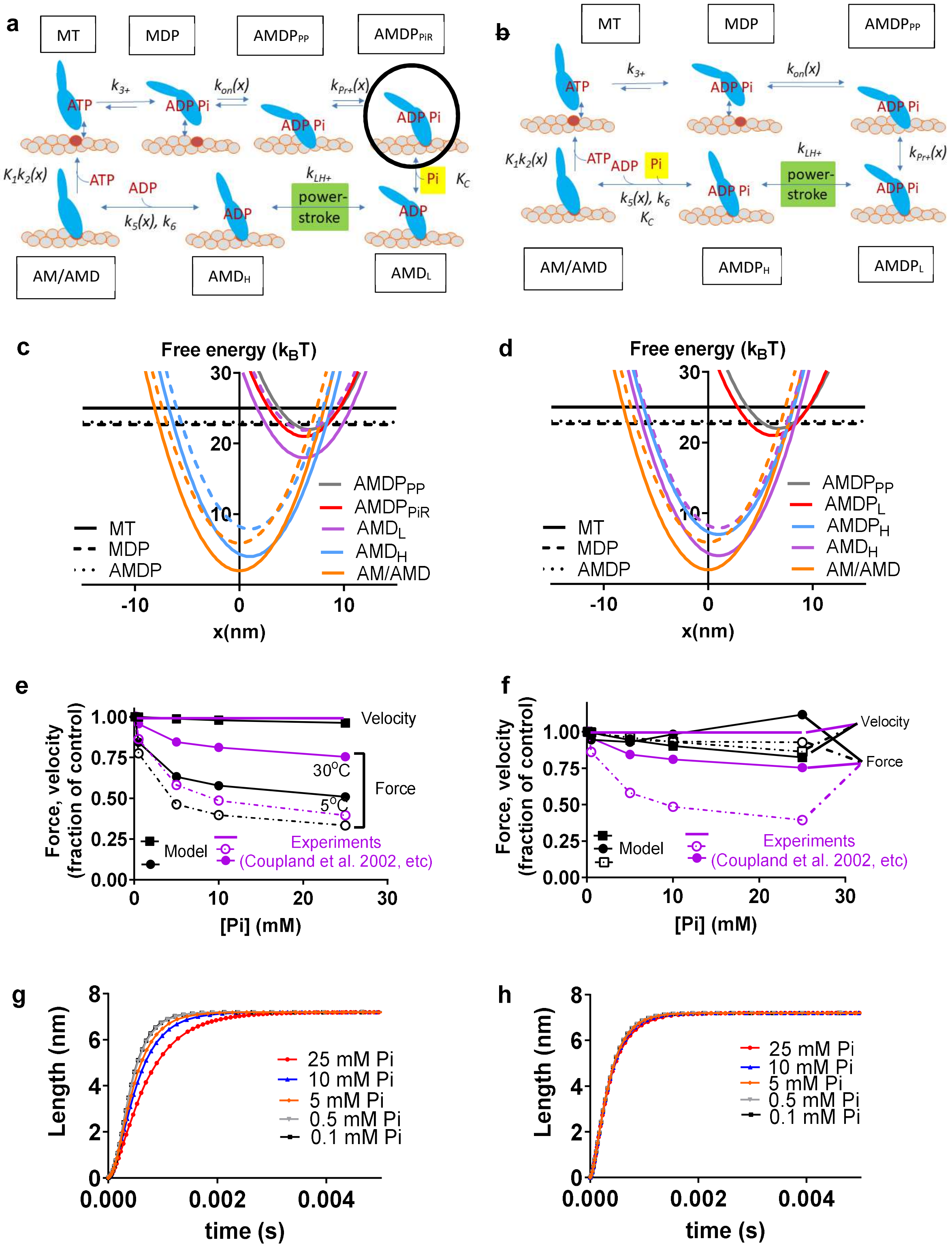

Supplementary Figure 6. Models with Pi-release before and after power-stroke including Pi-release state. Models developed from those in Supplementary Figure 5 by

inclusion of a weakly bound Pi-release state (AMD_{PiR}; encircled)[18] between the initial pre-power-stroke attachment state AMD_{PP} and the more strongly bound AMD_L state. **a** Cartoon illustrating the model with Pi-release before the power-stroke, modified from those in [11,18]. **b** Cartoon illustrating kinetic scheme for model modified from that in a to have Pi-release after the power-stroke. **c** Free energy diagrams for different cross-bridge states of model in a at 0.5 mM Pi (coloured full lines) and 25 mM Pi (coloured dashed lines). **d** Free energy diagrams for different cross-bridge states of model in b at 0.5 mM Pi and 25 mM Pi. **e** Simulated steady-state values for maximum isometric force (black circles) and maximum velocity of shortening (black squares) using the model depicted in a, compared to experimental data for velocity (e.g. [14,15]) and force [16] at 30 °C and 5 °C. **f** Data as in e but for model depicted in b, Note, that this displays wrong trend for simulated results vs [Pi] unlike the case in e. **g** Simulation using model in a and c of power-strokes (nm displacement vs time) for an ensemble of myosin heads attaching in the pre-power stroke actomyosin state with ADP and Pi at the active site and cross-bridge force clamped to 0 pN. **h** Simulations as in g but for model in b and d. Model parameter values for 30 °C and fast skeletal muscle myosin close to physiological ionic strength are given in Supplementary Tables 5 - 6. Parameter values for 5 °C indicated in Supplementary Table 7.

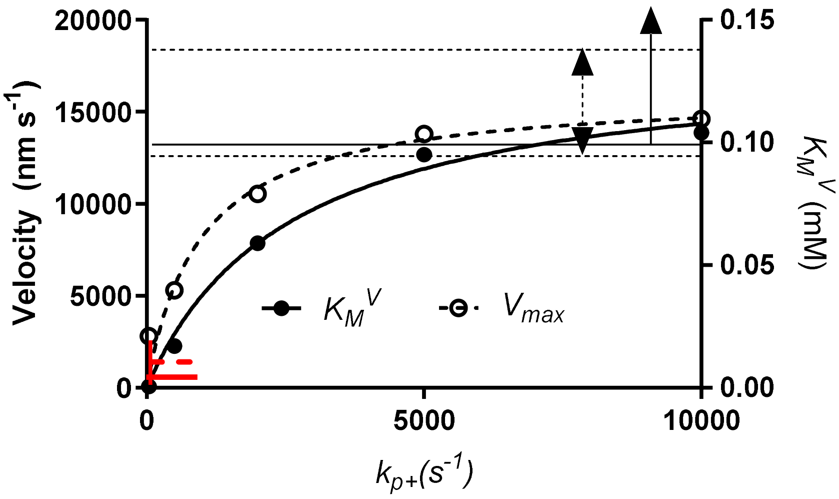

Supplementary Figure 7. Maximum sliding velocity at saturating [MgATP] (left vertical axis, dashed black curve) and the MgATP concentration (K_M^V; right vertical axis, black full curve) for half maximal velocity as function of the rate constant of Pi-release (k_{P+}) in model with Pi-release after the power-stroke from Supplementary Figure 6b. Experimentally observed range for maximum velocity at 30 °C indicated by dashed horizontal black lines and dashed double arrow[19-21]. The experimental lower limit for K_M^V indicated by full horizontal black line [22]. Full line arrow indicates that K_M^V values as high as 0.4 mM have been observed experimentally [23]. Note, if the rate constant for Pi release is as low (vertical red line) as found recently [8], both V_{max} (dashed horizontal red line) and K_M^V(full horizontal red line) are greatly underestimated by the model in Supplementary Figure 6b. The order of magnitude lower value of K_M^V predicted by the model in Supplementary Figure 6b than seen in experiments means that the model predicts orders of magnitude too low sensitivity of velocity to a reduction in [MgATP] from the physiological value (5 mM). Curved lines represent fits of rectangular hyperbola to the data to guide the eye.

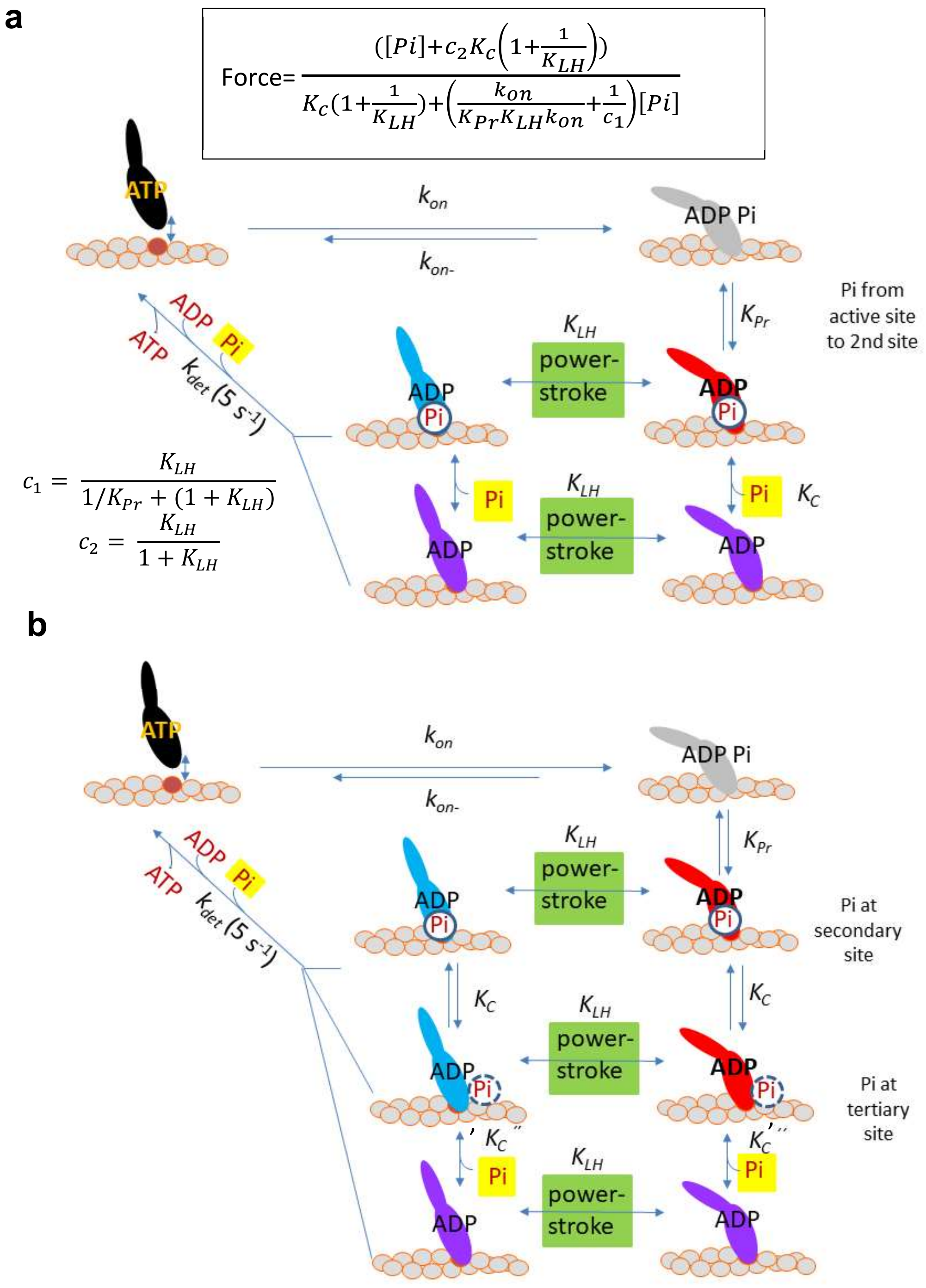
a
Force= $\frac{([Pi]+c_2K_c(1+\frac{1}{K_{LH}}))}{K_c(1+\frac{1}{K_{LH}})+(\frac{k_{on}}{K_{Pr}K_{LH}kon}+\frac{1}{c_1})[Pi]}$
ATP
k_{on}
k_{on-}
ADP Pi
K_{Pr}
Pi from active site to 2nd site
ADP
Pi
ATP
k_{det} (5 s-1)
K_{LH}
power-stroke
ADP
Pi
K_C
$c_1 = \frac{K_{LH}}{1/K_{Pr} + (1+K_{LH})}$
$c_2 = \frac{K_{LH}}{1+K_{LH}}$
b
ATP
k_{on}
k_{on-}
ADP Pi
K_{Pr}
ADP
Pi
ATP
k_{det} (5 s-1)
K_{LH}
power-stroke
Pi at secondary site
K_C
Pi at tertiary site
K_C''
Pi
ADP

Supplementary Figure 8. Versions of model in Fig. 4a-b of main text used for simulation of contraction under isometric conditions. Both models are derived by lumping together the detached states in Fig. 4 into one, integrating the AM/AMD state (orange in main Fig. 4) into the AMD_H states (post-power stroke purple and blue states) and assuming one constant cross-bridge strain for each state. As a further simplification, the force in the post-power-stroke AMD_H states is set to 1 whereas the force in all other states is set to 0. **a** A version of the model most similar to that in main Fig. 4a and the rate constants are approximated from that model and Supplementary Tables 5 - 6 assuming a strain of $x \approx x_1$ to: k_{on}=260 s^{-1} ; k_{on-}=130 s^{-1}; $K_{Pr}=k_{pr+}/k_{pr-}$; $k_{pr+}=k_{pr-}$=2000 s^{-1}; $K_{LH}=k_{LH+}/k_{LH-}$; k_{LH+}= 5000 s^{-1}; k_{LH-}=2500 s^{-1}. Finally, $K_C=k_{P+}/k_{P-}$=10 mM, k_{P+}=100 s^{-1} and k_{det}=5 s^{-1}. On the assumption that k_{det}=0 the isometric steady-state force as a function of [Pi] is given by the expression in the box (with constants c_1 and c_2 given to the left). This expression gives a force-[Pi] relationship that is negligibly different from that obtained by numerical solution of the differential equations with k_{det}=5 s^{-1} (plotted in main Fig. 4c). **b** Version of model in a, modified to include a tertiary Pi-binding site (electrostatic binding to myosin head surface) for which Pi-binding is assumed to be a rapid equilibrium governed by the dissociation constant K_C''= 20 mM. Furthermore, in this model $K_C' = k_{P+}/k_{P-}$ =0.286 with k_{p+} still set to 100 s^{-1}. The expression in the box in a is approximately valid also for the model version in b if the constant K_C is substituted for $K_C' K_C''$.

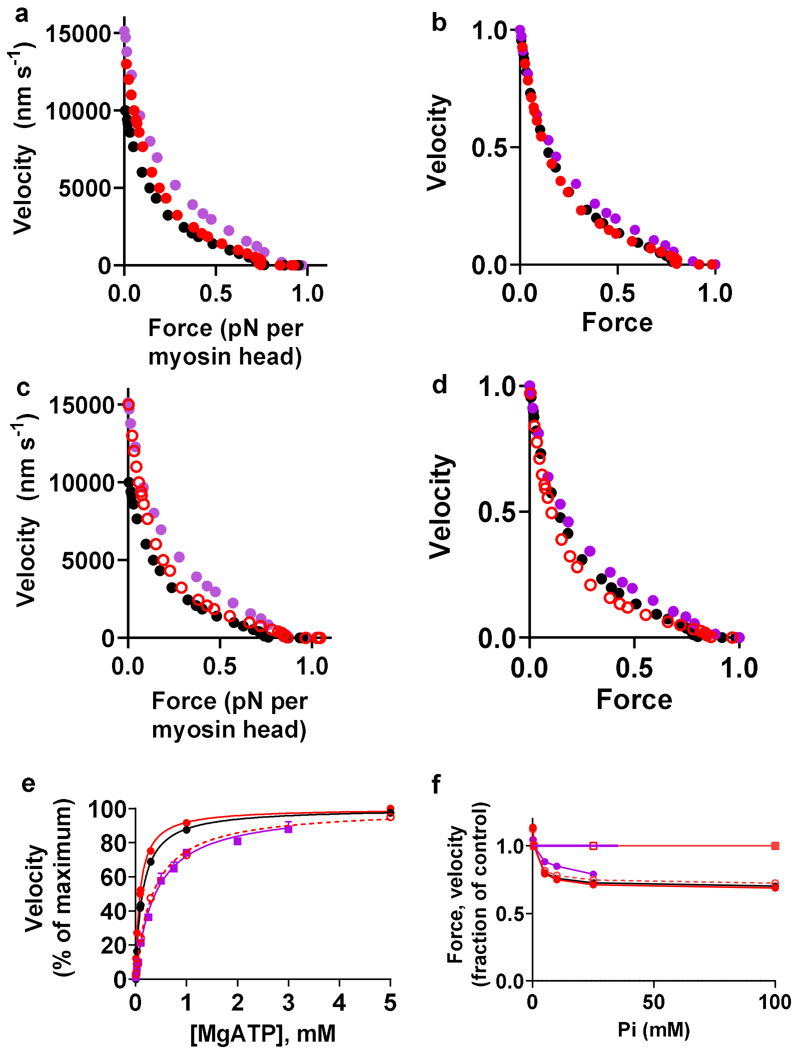

Supplementary Figure 9. The relationship between [Pi] on the one hand and force and velocity on the other is robust to changes in parameter values. a Prediction of force-velocity data for model in Fig. 4a, b for standard parameter values (black) and modified parameter values (red): k_{Pr+}'=10000 s^{-1}; k_2=2500 s^{-1}, x_1=-0.5 nm, x_{11}=0 nm compared to experimental data[51] (purple) scaled for maximum isometric force to coincide with the simulated value. **b** Data in a replotted after scaling of force to the maximum isometric force and the velocity to maximum velocity. **c** Prediction of force-velocity data for the model shown in Fig. 4a,b for standard parameter values (black) and modified parameter values (red): k_{Pr+}'=10000 s^{-1} and non-linear cross-bridge elasticity (as in Supplementary Figure 10) compared to experimental data [51] (purple) as in a. **d** Data in c replotted after scaling of force to the maximum isometric force and velocity to maximum velocity. **e** Velocity vs [MgATP] predicted by the model in Fig. 4 using standard parameter values (black), modified parameter values as in a and b (filled red circles full line), modified parameter values from c, d (open red circles, dashed line) and experimental data [52] (purple squares). The curves represent rectangular hyperbola fitted to the data with [MgATP]-values (K_M^v) for half-maximum

velocity (mean ± 95 % CI): 0.138 ± 0.03 mM (black), 0.387 ± 0.03 mM (purple). **f** Isometric force vs [Pi] depicted using colour coding corresponding to that in e. Note, **i.** the absolute value of the maximum velocity of shortening is not correctly predicted using our standard set of parameter values (black; Supplementary Tables 5 - 6), **ii.** the model reproduces the relationship between velocity and [MgATP] as faithfully as previously shown for the models in Supplementary Figures. 5 - 6 [18,19], but like these, with lower [MgATP] (K_M^V) for half-maximum velocity than found experimentally. Interestingly, predictions of both the maximum velocity and K_M^V is greatly improved if the rate constant k_{Pr+} (poorly characterized experimentally) is increased several-fold and if non-linear elasticity (a debated feature)[53,54] is assumed for the AM/AMD state (cf. [52,53]). The effects of these changes and/or some other modifications of model parameters within experimental uncertainties are depicted by red symbols in this figure. Remarkably, the key effects of [Pi] on velocity and force are robust to these modifications of model parameters (this figure; panel f).

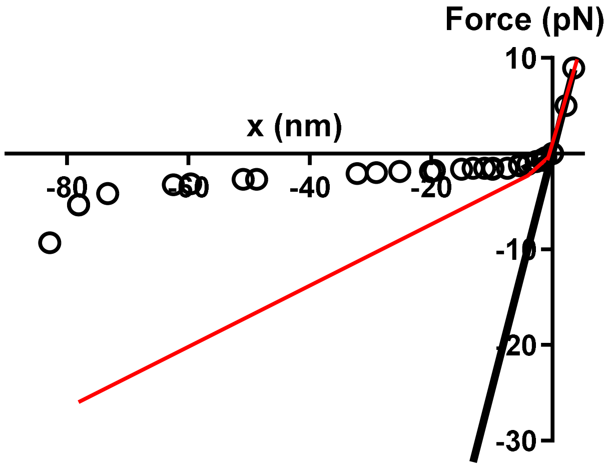

Supplementary Figure 10. Cross-bridge force vs strain in the AM/AMD state with linear and non-linear cross-bridge elasticity. Circles: data measured from Fig. 1 of Kaya and Higuchi[1]. Black full line: Linear cross-bridge elasticity. Red full line: Force-strain relationship for non-linear cross-bridge elasticity used here as compromise (cf. [24]) between the case with linear elasticity and the non-linearity found by Kaya and Higuchi[1].

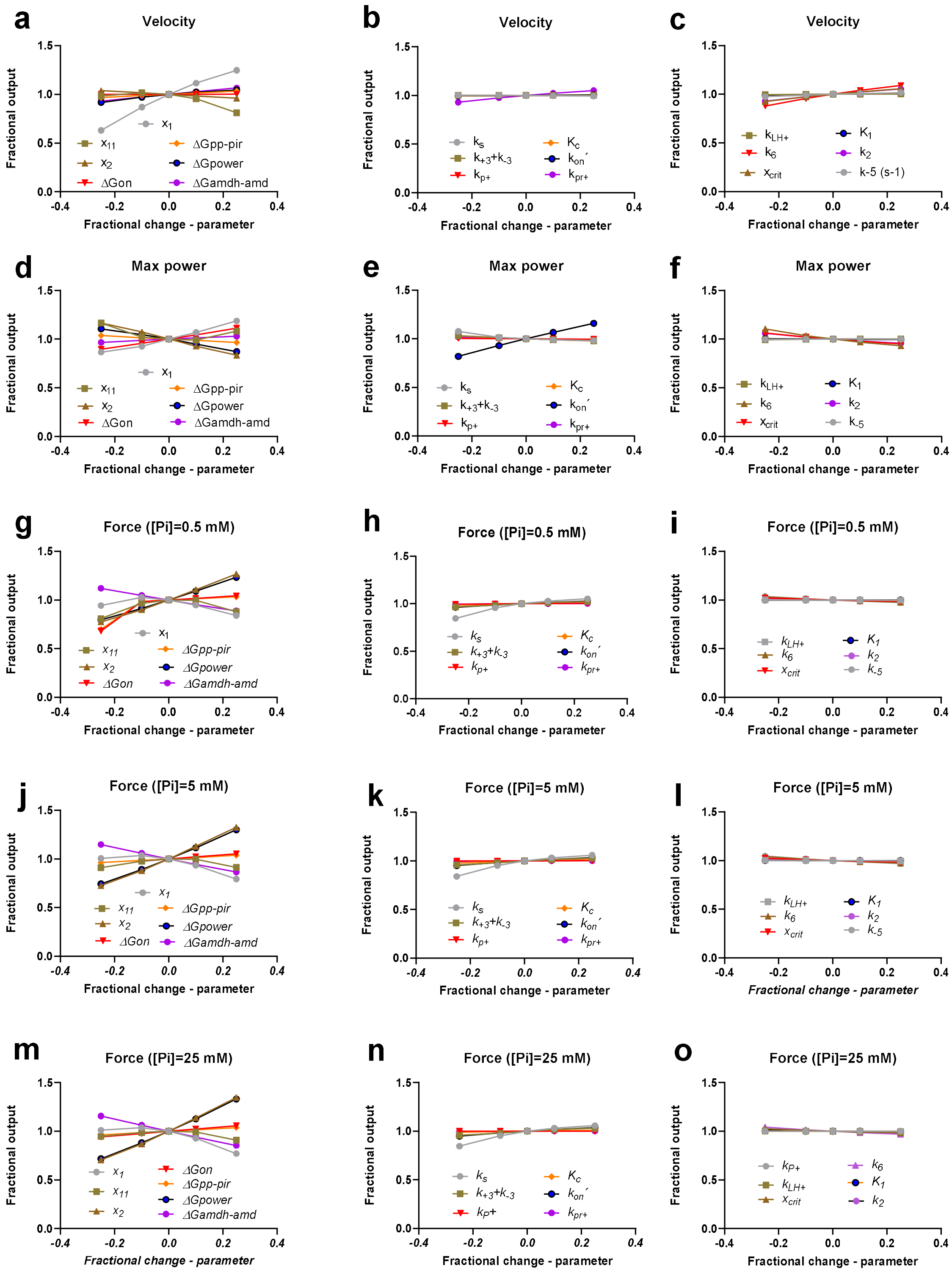

Supplementary Figure 11. Sensitivity analysis for the model in main Fig. 4. (a-c) Simulated velocity. **(d-f)** Simulated maximum power at [Pi]=0.5 mM; normalized to product of maximum velocity and maximum isometric force. **(g-o)** Simulated maximum isometric force (at different [Pi]). $\Delta G_{power} \equiv \Delta G_{AMD(P)L\text{-}AMD(P)H}$ in Supplementary Table 5.

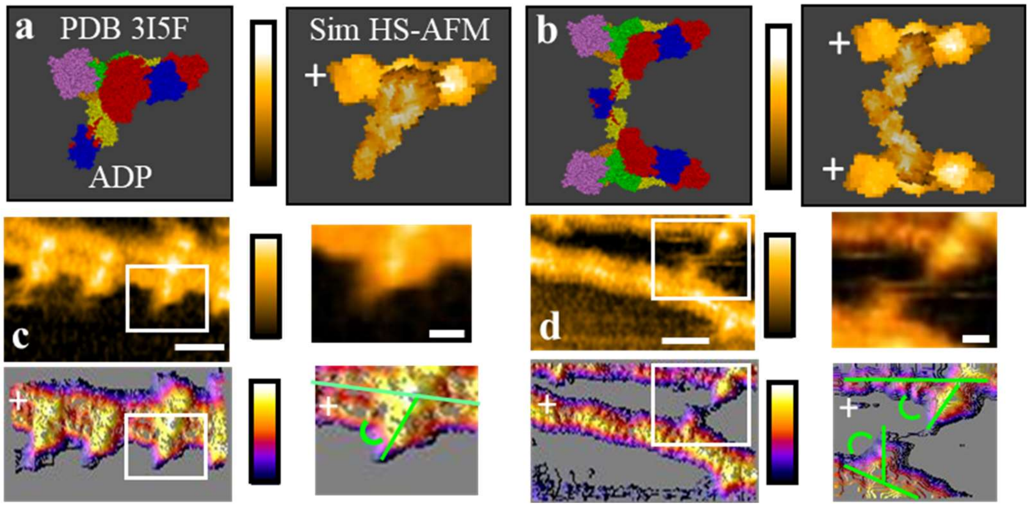

Supplementary Figure 12. ADP-lever arm position in the actin-myosin complex. a-b Atomic models and corresponding simulated hs-AFM images of the actin-myosin S1-ADP complex (PDB 2Y83, PDB 3I5F) for two experimental approaches used in this study. Two heads attached to a given actin filament in **a** and to different actin filaments in **b**. Simulated hs-AFM images were obtained by Bio-AFM viewer software (v.2.0). **c-d** The ADP- lever arm position of myosin relative to actin filament, when the HMM heads were bound to one actin filament in **c** and when each head was bound to a different, nearly parallel actin filaments in **d**. The ADP-lever arms and myosin heads are highlighted by the 3D-filtered images and the angle measurement of lever arm position relative to actin filament is indicated by green lines. The scale bars are 30 nm for non-zoomed images and 10 nm for zoomed images.

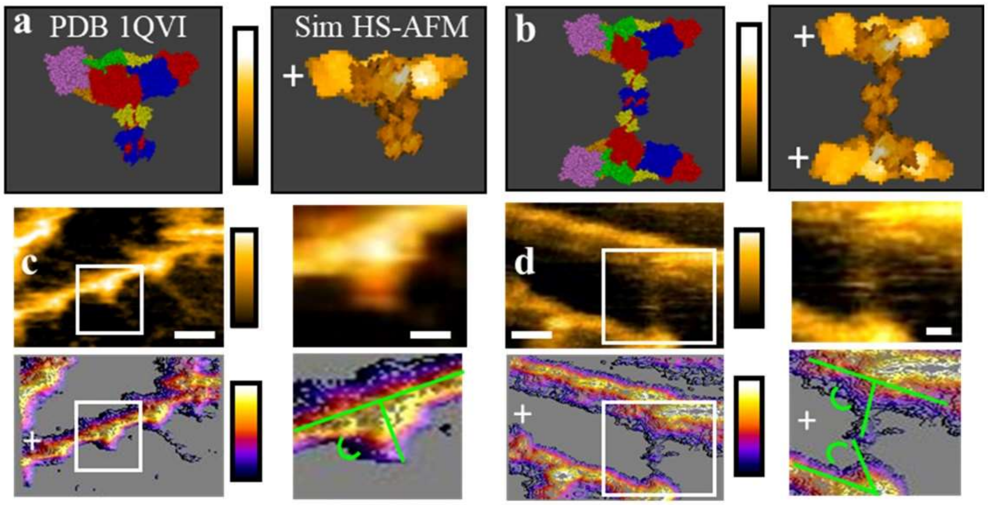

Supplementary Figure 13. ADP-vanadate lever arm position in the actin-myosin complex. a-b Atomic models and corresponding simulated hs-AFM images of the actin-myosin S1-ADP-vanadate complex (PDB 2Y83, PDB 1QVI) for two experimental approaches used in this study. Simulated hs-AFM images were obtained by Bio-AFM viewer software (v.2.0). **c-d** The ADP-vanadate lever arm position of myosin relative to actin filament, when the HMM heads were bound to one actin filament in **c** and when each head was bound to the two parallel actin filaments in **d**. Zoomed images to the right in each panel. The ADP-vanadate lever arms and myosin heads are highlighted by the 3D-filtered images and the angle measurements of lever arm positions relative to actin filament are indicated by green lines. The scale bars are 30 nm for non-zoomed images and 10 nm for zoomed images.

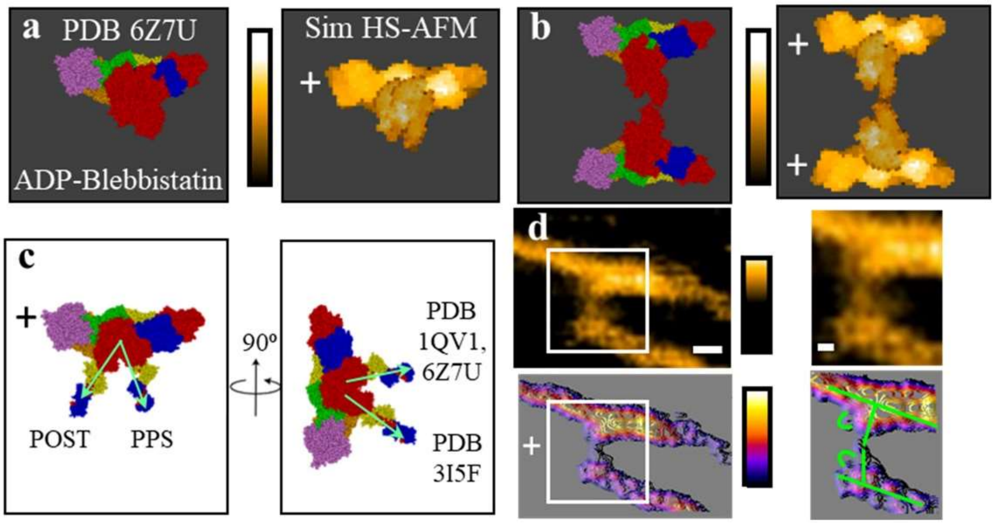

Supplementary Figure 14. ADP-blebbistatin lever arm position in the actin-myosin complex. **a-b** Atomic models and corresponding simulated hs-AFM images of the actin-myosin S1-blebbistatin complex (PDB 2Y83, PDB 6Z7U) in the pre-power stroke state. Simulated hs-AFM images were obtained by Bio-AFM viewer software (v.2.0). **c** The difference in lever arm position during the power stroke comparing pre-power-stroke position (PPS) and post-power-stroke position (POST). **d** hs-AFM snapshots of myosin heads binding to the two parallel actin filaments in pre-power-stroke positions with zoom in of area within box to the right. The ADP-blebbistatin lever arms and myosin heads are highlighted by the 3D-filtered images and the angle measurement of lever arm position relative to actin filament is indicated by green lines. The scale bars are 30 nm for non-zoomed images and 10 nm for zoomed images.

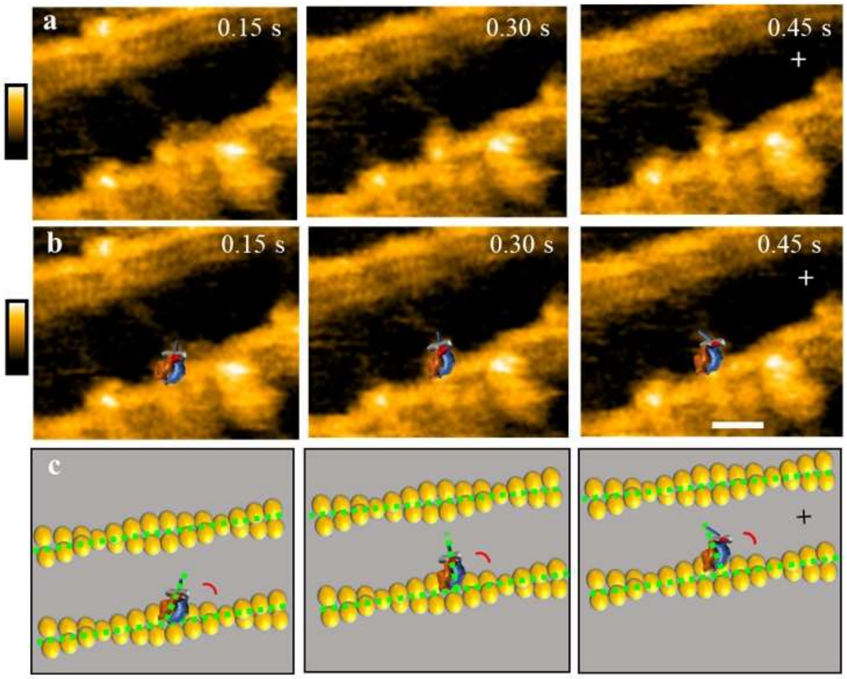

Supplementary Figure 15. Measurement of the lever arm position in the actin-myosin complex. a hs-AFM successive images of actin-myosin-ADP-vanadate complex with myosin heads bound to the two parallel actin filaments and **b, c** corresponding lever arm and heads orientations used to calculate the angle of the lever arm. The transition from the strong, post-power-stroke (0.15 s), to the weak pre-power-stroke (0.30-0.45 s) lever arm positions of the lower myosin head is depicted. Scanning rate 6.7 fps for a scan area of 150 x 120 nm^2 with 80 x 64 $pixels^2$. Scale bar 30 nm.

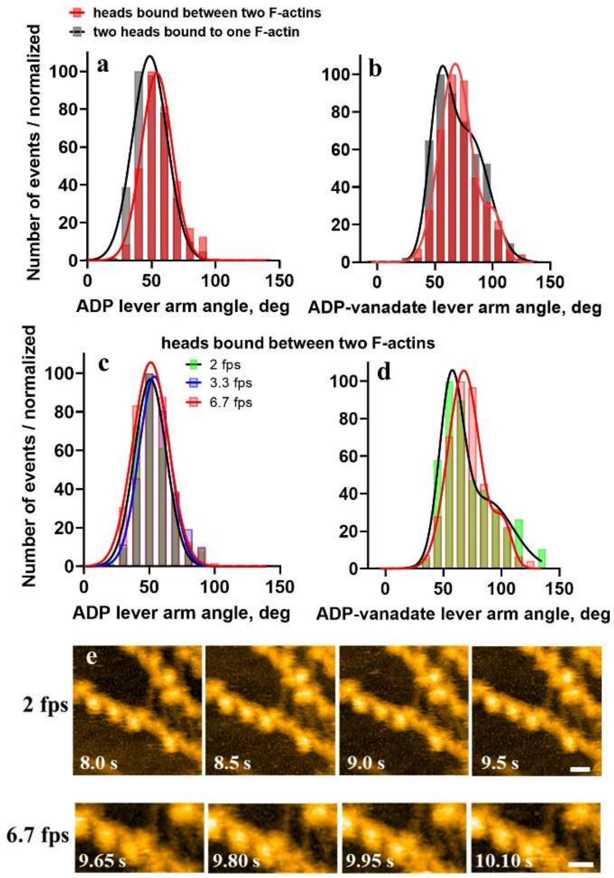

Supplementary Figure 16. The distributions of the lever arm angles obtained at different scan speeds and by using two experimental approaches for binding HMM heads to actin filaments. **a** lever arm distribution in the presence of ADP for the two types of HMM heads binding (number of events: 597 vs. 519); **b** Lever arm distribution in the presence of ADP-metavanadate for the two types of HMM heads binding (number of events: 504 vs. 190). Statistical hypothesis tests for differences between methods: ADP: p=0.81, unpaired two-sided t-test; ADP-metavanadate: p = 0.38, unpaired two-sided t-test; **c** lever arm distribution in the presence of ADP for different frame rates; 6.7 fps: 659 events (51.0 ° ± 13.7). 3.3 fps: 268 events (53.9 ° ± 11.8). 2 fps: 189 events (50.8 ° ± 12.2), p = 0.97, 1way ANOVA); **d** lever arm distribution in the presence of ADP-vanadate for different frame rates (number of events: 504 vs. 82), p = 0.92, unpaired two-sided t-test, n = 3 independent experiments; **e** Binding of HMM heads to two parallel actin filaments in the presence of ADP at 2 fps for a scan area of 200 x 200 nm^2 with 120 x 120 pixels^2 and 6.7 fps for a scan area of 150 x 75 nm^2 with 80 x 40 pixels^2. Scale bars: 30 nm.

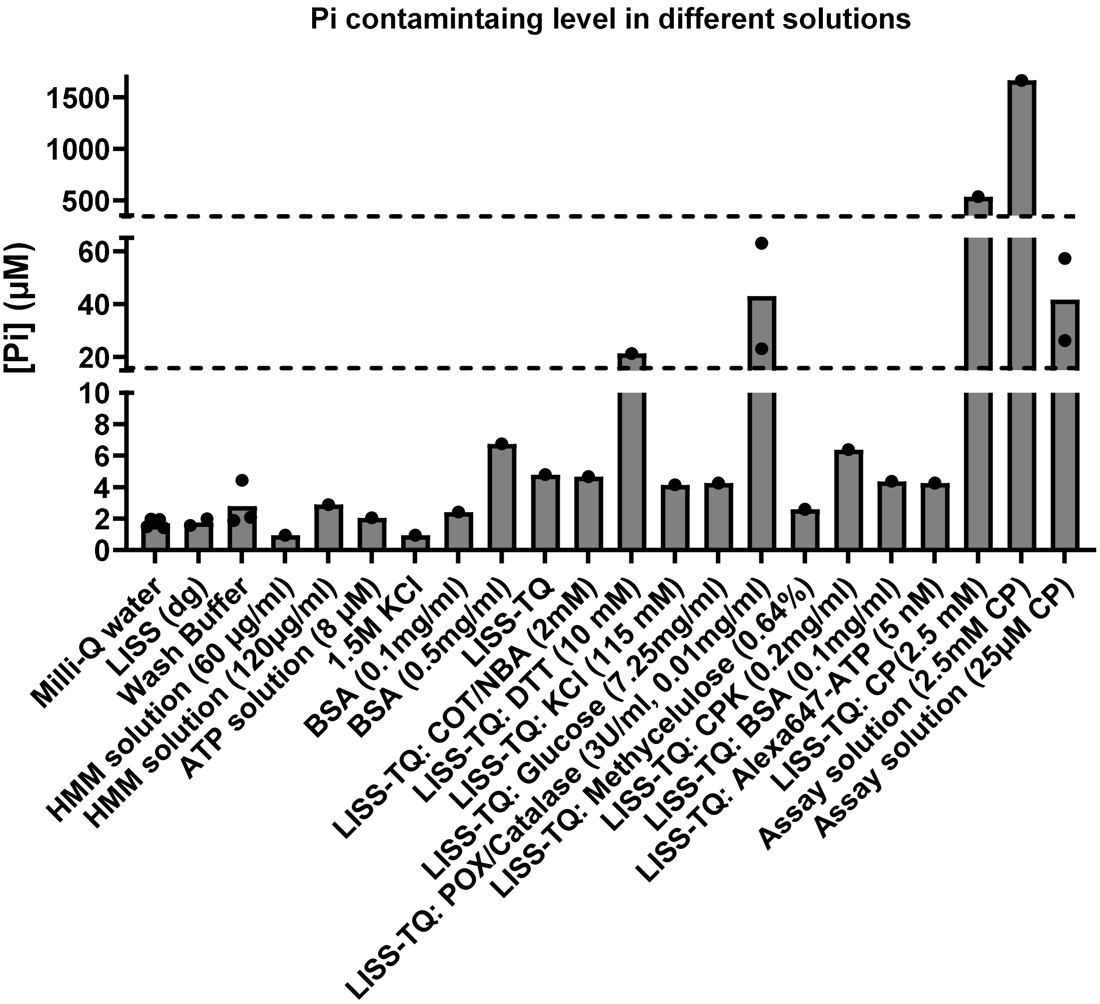

Supplementary Figure 17: Pi contaminating level in different solutions as measured using Phosphate assay Kit. LISS: low ionic strength solution (pH 7.4) contained 10 mM MOPS, 1 mM $MgCl_2$ and 0.1 mM K_2EGTA. Wash buffer: 50 mM KCl and 1 mM DTT in LISS. LISS-TQ: Trolox-Trolox/Trolox Quinone mixture in LISS. All the further ingredients were diluted in that buffer. COT/NBA: cyclooctatetraene/4-Nitrobenzyl alcohol. DTT: dithiothreitol. POX: pyranose oxidase. CPK: creatine phosphokinase. CP: creatine phosphate. Note substantial reduction in whole assay buffer contaminating Pi (from 1.6 mM to ≤60 µM) once the creatine phosphate was reduced from standard 2.5 mM to 25µM). Individual measurements are shown. Where possible mean was calculated.

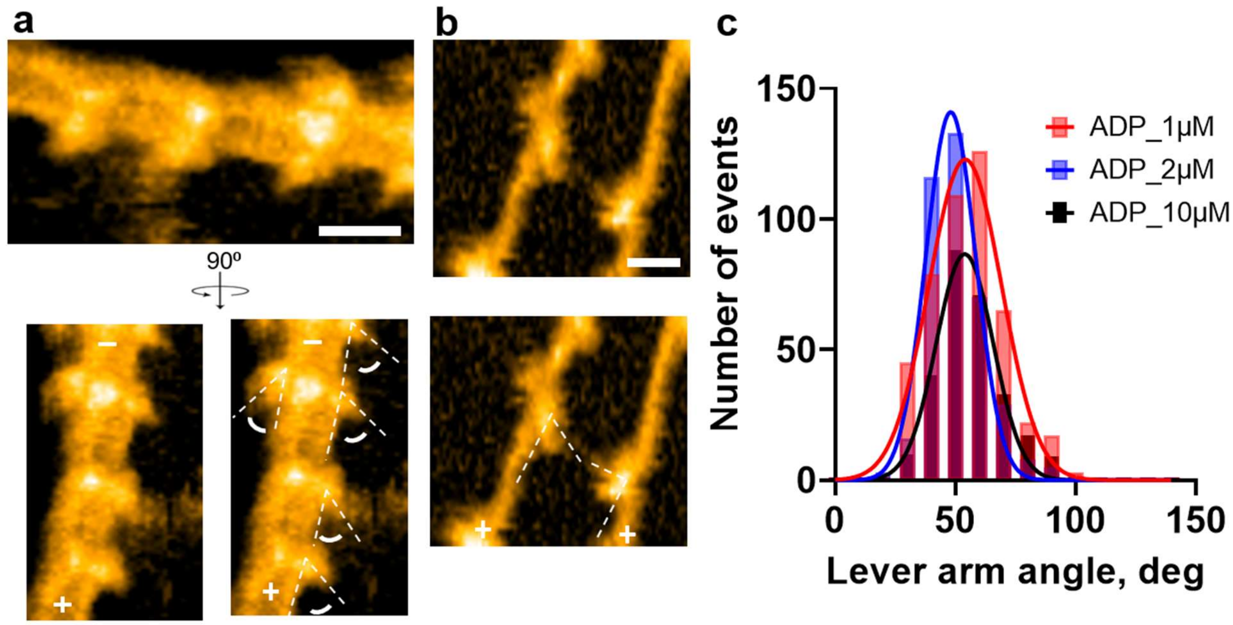

Supplementary Figure 18. The polarity of the actin filaments observed by orientation of the HMM heads bound to one actin filament or to two different actin filaments in the presence of ADP. **a-b** HMM molecules bound to actin filaments in the presence of 1 μM **a** or 10 μM **b** ADP. The polarity of actin filaments evaluated by the orientation of the HMM heads and lever arms, directed toward to the barbed (+) end. It is easier to visualize after 90° rotations as shown in lower left panels. Scan area: 150x75 nm^2, 80x40 $pixels^2$, scale bars: 30 nm. **c** lever arm configuration in the presence of 1 μM, 2 μM and 10 μM ADP. Note the similarity in the lever arm angle between evaluated ADP concentrations. The mean values (±SD) obtained from Gaussian distributions - 1 μM ADP: 54.06 ± 15.13, r^2=0.98 (468 events); 2 μM ADP: 48.04 ± 10.40, r^2=0.98 (380 events); 10 μM ADP: 53.87 ± 11.84, r^2=0.98 (268 events), n = 3 independent experiments. The imaging was performed in the presence of 10 mM glucose and 1 U/ml of hexokinase to remove possible presence of ATP in the ADP solutions.

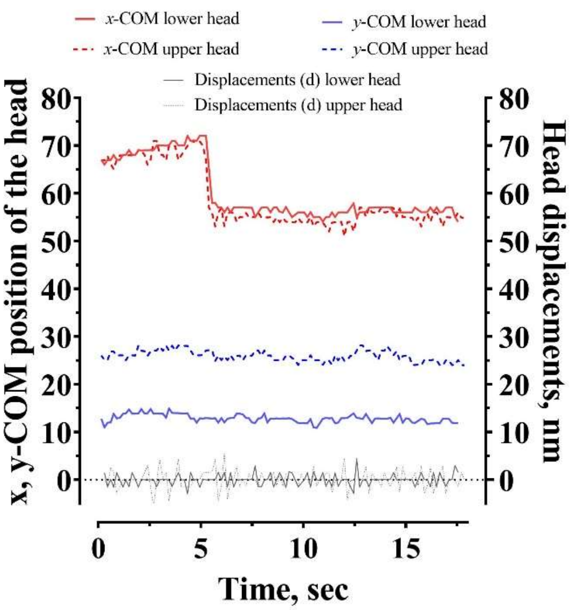

Supplementary Figure 19. The drift analysis of the system with x, y- center of mass (COM) position of lower and upper HMM heads bound to two different actin filaments in the nucleotide-free state combined with the displacement analysis of each head in time. Note, that a decrease of the COM x-position after ~5 sec was due to manual change of the sample stage to place the HMM molecule bound to parallel actin filaments at the center of view as can be seen in the Movie S11 (5th movie panel). In all buffers and HMM preparations used for the nucleotide-free experiments, 1 U/ml of apyrase was added to remove any traces of ADP or ATP contaminants. The displacements analysis of the lower and upper head displacements calculated as a difference between the center of mass (COM) values of the head at the reference frame and the head COM values in the next frame. The data for the drift and displacement analyses are included in the hs-AFM Source Data File.

Supplementary Tables

Supplementary Table 1. Summary of best mean values ± 95% CI (Error) from exponential fits for data in Figure 2c related to bar diagrams in Figure 2d and the diagram in Figure 2e

**Rates**													
$[Pi]_{added}$ (mM)		$k_{unsp. fast}$ (s^{-1})			$k_{unsp. slow}$ (s^{-1})			k_{ATPase} (s^{-1})			$k_{ATPase2}$ (s^{-1})*		
		Mean	+Error	-Error	Mean	+Error	-Error	Mean	+Error	-Error	Mean	+Error	-Error
0		2.874	0.0885	0.086	0.3199	0.0063	0.0062	0.0512	0.0004	0.0004			
		2.5616	0.0611	0.0596	0.2994	0.0064	0.0063	0.0478	0.0009	0.0009			
0.2		2.5652	0.0744	0.072	0.2562	0.0061	0.0059	0.0472	0.0014	0.0014			
0.3		2.8429	0.1209	0.116	0.3364	0.0101	0.0099	0.0599	0.0005	0.0005			
		1.6638	0.0762	0.0715	0.284	0.0148	0.0136	0.0611	0.0075	0.0076			
0.7		3.9086	0.2741	0.2409	0.6797	0.0248	0.0237	0.057	0.0005	0.0005			
1.3		3.2708	0.1481	0.1408	0.3164	0.0376	0.0322	0.0665	0.0076	0.0079			
					0.8594	0.0197	0.019	0.0926	0.0041	0.0039			
2.7		2.2533	0.2782	0.2522	0.4559	0.0279	0.0289	0.1138	0.0041	0.0044			
5.4		3.7096	0.1451	0.1408	0.5156	0.0124	0.0123	0.0569	0.0009	0.0009			
		3.2012	0.1318	0.1272				0.1249	0.001	0.001	0.0053	0.0021	0.0021
10.8		2.0302	0.1075	0.1027				0.0697	0.0019	0.0018			
21.5		4.0417	0.1551	0.1499				0.1654	0.0033	0.0033			
								0.0571	0.0037	0.0036			
43		4.5839	0.439	0.4054									
					0.6361	0.0575	0.0535						
43_{BSA}		3.8229	0.2101	0.2									
					0.7772	0.0541	0.0509						

**Amplitudes**														
$[Pi]_{added}$ (mM)	Events (N)	$A_{unsp.\ fast}$ (%)			$A_{unsp.\ slow}$ (%)			A_{ATPase} (%)			$A_{ATPase2}$ (%)*		
		Mean	+Error	-Error	Mean	+Error	-Error	Mean	+Error	-Error	Mean	+Error	-Error
0	754	34.566	0.4785	0.4815	37.433	0.7572	0.7615	28.001	0.2787	0.2801			
	374	48.005	0.5552	0.5612	38.887	1.4131	1.4126	13.108	0.3247	0.323			
0.2	410	49.838	0.5393	0.5464	39.938	0.9611	0.9603	10.224	0.4218	0.4138			
0.3	572	35.595	0.6116	0.6146	34.07	0.9835	0.9916	30.335	0.372	0.377			
	287	47.479	1.4339	1.4753	45.505	9.8182	9.0466	7.0154	1.3018	1.1767			
0.7	423	31.662	1.4293	1.4899	39.445	1.7838	1.8431	28.893	0.3546	0.3531			
1.3	199	61.434	1.294	1.3864	29.13	3.035	3.0755	9.4353	1.741	1.6891			
	130				85.386	0.6309	0.653	14.614	0.6309	0.653			
2.7	203	23.581	2.4181	2.1651	52.179	3.9999	3.8518	24.24	1.5818	1.6867			
5.4	355	42.372	0.9206	0.9243	42.655	1.2024	1.2044	14.973	0.2817	0.2801			
	230	33.796	0.5781	0.5849				64.9886	0.74333	0.73438	1.2151	0.1653	0.1495
10.8	116	74.921	0.5774	0.5925				25.079	0.5774	0.5925			
21.5	99	69.307	0.6075	0.621				30.693	0.6075	0.621			
	10							100					
43	16	100											
	13				100								
43_{BSA}	38	100											
	18				100								

* not shown in Figure 2d and e due to invisibility on graphs because of very low values. The ATPase2 phase refers to a very slow phase of ATP turnover presumably related to surface adsorption of myosin head. See ref (7) for details.

Supplementary Table 2. Summary of best mean ± 95% CI (Error) parameter values of exponential fits for data in Figure 3c related to bar diagrams in Figure 3d and the diagram in Figure 3e

Rates										
Experimental condition		$k_{unsp.\,fast}$ (s^{-1})			$k_{unsp.\,slow}$ (s^{-1})			k_{ATPase} (s^{-1})		
		Mean	+Error	-Error	Mean	+Error	-Error	Mean	+Error	-Error
HMM		2.085	0.0968	0.0933	0.2179	0.0021	0.0021	0.0323	0.0001	0.0001
HMM*D•Vi		1.4556	0.0275	0.0268	0.2131	0.0024	0.0023	0.0332	0.0008	0.0008
HMM*D•Vi @ $[Pi]_{added}$= 43mM		3.835	0.6243	0.5605				0.0848	0.0078	0.0078
BSA @ $[Pi]_{added}$= 43mM		4.2491	1.6408	1.1541	0.3987	0.0119	0.0122			
**Amplitudes**										
	Events (N)	$A_{unsp.\,fast}$ (%)			$A_{unsp.\,slow}$ (%)			A_{ATPase} (%)		
		Mean	+Error	-Error	Mean	+Error	-Error	Mean	+Error	-Error
HMM	1233	17.781	0.3463	0.3476	49.101	0.5258	0.5267	33.118	0.1795	0.1791
HMM*D•Vi	776	43.38	0.4062	0.4068	50.851	0.5907	0.5864	5.7691	0.1845	0.1797
HMM*D•Vi @ $[Pi]_{added}$= 43mM	11	67.032	2.1537	2.342				32.968	2.1537	2.342
BSA @ $[Pi]_{added}$= 43mM	37	18.702	3.2548	3.4267	81.298	3.2548	3.4267			

Supplementary Table 3. Parameter values[a] for models in Supplementary Figures 5a and b, determining shape of free energy diagrams for simulation of contractile properties of fast mammalian muscle at 30 °C

**Parameter[b]**	**Model Pi release before power-stroke**	**Model Pi release after power-stroke**
x_1 (AMDP$_{PiR}$, AMD$_L$)	7.7 nm	7.7 nm
x_2 (AMD$_H$)	1.0 nm	1.0 nm
x_3	0 nm	0
ΔG_w*(MDP- AMDP)*	2.5 k_BT	2.5 k_BT
ΔG_{on} *(AMDP –AMDP$_T$ or AMDP –AMDP$_L$)*	0.7 k_BT	0.7 k_BT
$\Delta G_{AMDPT\text{-}AMDL}$ (AMDP$_T$ – AMD$_L$)	$k_BT \ln([P_i]/K_C)$	-
$\Delta G_{AMD(P)L\text{-}AMD(P)H}$ (AMD(P)$_L$- AMD(P)$_H$)	13 k_BT	13 k_BT
$\Delta G_{AMDPH\text{-}AMD(P)H}$ (AMDP$_H$- AMD$_H$)	-	$k_BT \ln([P_i]/K_C)$
$\Delta G_{AMDH\text{-}AMD}$ (AMD$_H$- AMD)	2 k_BT	2 k_BT
ΔG_{ATP}	13.1 + ln ([MgATP]/ ([MgADP][Pi]) k_BT	13.1 + ln ([MgATP]/ ([MgADP][Pi]) k_BT
k_S	2.8 pN/nm	2.8 pN/nm

Footnotes to Supplementary Table 3
NA: Not applicable; IS: ionic strength
[a] The parameter values were from two-headed myosin motor fragments from fast skeletal muscle of rabbit at 30°C, ionic strength 130-200 mM, pH 7-8. For further details, see [12,18,26].
[b] The states most related to the parameter given in parentheses

Supplementary Table 4. Parameter values[a] for models in Supplementary Figures 5a and b, defining rate functions and kinetic constants for simulation of contractile properties of fast mammalian muscle at 30 °C

**Parameter**	**Model, Pi release before power-stroke**	**Model, Pi release after power-stroke**
$k_{+3} + k_{-3}$	220 s^{-1}	220 s^{-1}
K_3	10	10
k_{-5}	2000 s^{-1}	2000 s^{-1}
Kc	10 mM	10 mM
k_{on}'	100 s^{-1}	100 s^{-1}
k_{P+}	10000 s^{-1}	10000 s^{-1}
k_{LH+}	6000 s^{-1}	6000 s^{-1}
x_{crit}	0.6 nm	0.6 nm
k_6	5000 s^{-1}	5000 s^{-1}
Physiological [Pi]	0.5 mM	0.5 mM
[MgATP]	5 mM	5 mM
K_1	1.7 mM^{-1}	1.7 mM^{-1}
k_2	2000 s^{-1}	2000 s^{-1}

[a] The parameter values were from two-headed myosin motor fragments from fast skeletal muscle of rabbit at 30°C, ionic strength 130-200 mM, pH 7-8. For further details, see [12,18,26].

Supplementary Table 5. Parameter values[a] for models in Supplementary Figures 6a, b and main Figs. 4-5 determining shape of free energy diagrams for simulation of contractile properties of fast mammalian muscle at 30 ºC[a]

Parameter	Model, Pi release before power-stroke	Model, Pi release after power-stroke	Model, Two-step Pi-release (Figs. 4-5)
x_{11} (AMDP, $AMDP_{PP}$)	7.2 nm	7.2 nm	7.2 nm
x_1 ($AMDP_{PiR}$, AMD_L)	6.7 nm	6.7 nm	6.7 nm
x_2 (AMD_H)	1.0 nm	1.0 nm	1.0 nm
x_3	0 nm	0 nm	0 nm
ΔG_w(MDP- AMDP)	0 k_BT	0 k_BT	0 k_BT
$\Delta G_{AMDP\text{-}AMDP\text{-}PP} \equiv \Delta G_{on}$ (AMDP – $AMDP_{PP}$)	0.7 k_BT	0.7 k_BT	0.7 k_BT
ΔG_{PiR} ($AMDP_{PP}$ – $AMDP_{PiR}$)	1 k_BT	-	-
$\Delta G_{AMDP\text{-}PiR\text{-}AMD\text{-}L}$ ($AMDP_{PiR}$ –AMD_L)	k_BT ln([P_i]/K_C)	-	-
$\Delta G_{AMDP\text{-}AMDL/AMDP'L}$ (AMDP – AMD_L/$AMDP'_L$)	NA	1 k_BT	1 k_BT
$\Delta G_{AMD(P)L\text{-}AMD(P)H}$ ($AMD(P)_L$-$AMD(P)_H$)	14 k_BT	14 k_BT	14 k_BT
$\Delta G_{AMDPH\text{-}AMDH}$ ($AMDP_H$-AMD_H)	-	k_BT ln([P_i]/K_C)	k_BT ln([P_i]/K_C)
$\Delta G_{AMDH\text{-}AMD}$ (AMD_H- AMD)	2 k_BT	2 k_BT	2 k_BT
ΔG_{ATP}	13.1 + ln ([MgATP]/ ([MgADP][Pi]) k_BT	13.1 + ln ([MgATP]/ ([MgADP][Pi]) k_BT	13.1 + ln ([MgATP]/ ([MgADP][Pi]) k_BT
k_s	2.8 pN/nm	2.8 pN/nm	2.8 pN/nm

The parameter values were from two-headed myosin motor fragments from fast skeletal muscle of rabbit at 30ºC, ionic strength 130-200 mM, pH 7-8. For further details, see [12,18,26].

Supplementary Table 6. Parameter values[a] for models in Supplementary Figures 6a, b, and main Figs. 4-5, defining rate functions and kinetic constants for simulation of contractile properties of fast mammalian muscle at 30 °C.

Parameter	Model, Pi release before power-stroke	Model, Pi release after power-stroke	Model, Two-step Pi-release (Figs. 4-5)
$k_{+3} + k_{-3}$	220 s^{-1}	220 s^{-1}	220 s^{-1}
K_3	10	10	10
k_{-5}	2000 s^{-1}	2000 s^{-1}	2000 s^{-1}
Kc	10 mM	10 mM	10 mM
k_{on}'	130 s^{-1}	130 s^{-1}	130 s^{-1}
k_{Pr+}'	3000 s^{-1} [b]	3000 s^{-1} [b]	3000 s^{-1} [b]
k_{P+}	10000 s^{-1}	10000 s^{-1}	100 s^{-1}
k_{LH+}	6000 s^{-1}	6000 s^{-1}	6000 s^{-1}
x_{crit}	0.6 nm	0.6 nm	0.6 nm
k_6	5000 s^{-1}	5000 s^{-1}	5000 s^{-1}
Physiological [Pi]	0.5 mM	0.5 mM	0.5 mM
[MgATP]	5 mM	5 mM	5 mM
K_1	1.7 mM^{-1}	1.7 mM^{-1}	1.7 mM^{-1}
k_2	2000 s^{-1}	2000 s^{-1}	2000 s^{-1}

[a] The parameter values were from two-headed myosin motor fragments from fast skeletal muscle of rabbit at 30°C, ionic strength 130-200 mM, pH 7-8 unless otherwise stated. For further details, see [12,18,26].

[b] Note, difference from model in [18] where the same parameter value was set to 1000 s^{-1} under control conditions. Here, a higher value was necessary to assume in order to achieve the experimentally observed maximum shortening velocity without changing other parameter values from their literature data.

Supplementary Table 7. Parameter values[a] for simulation of contractile properties of fast mammalian muscle at 5 °C using models in Supplementary Figures 6a, b and Fig. 4.

Parameter	Model, Pi release before power-stroke	Model, Pi release after power-stroke	Model, Two-step Pi-release (Fig. 4)
$\Delta G_{AM^*DL-AM'DH} \equiv \Delta \boldsymbol{G_{LH}}$ (AM*D_L-AM*D_H)	6 k_BT	6 k_BT	6 k_BT
$k_{+3} + k_{-3}$ (Recovery stroke+hydrolysis)	12.5 s^{-1}	12.5 s^{-1}	12.5 s^{-1}
K_3	4	4	4
k_{on}'	25 s^{-1}	25 s^{-1}	25 s^{-1}
k_2	413 s^{-1}	413 s^{-1}	413 s^{-1}
k_{P+}'	750 s^{-1}	750 s^{-1}	750 s^{-1}

[a]Parameter values not given here are assumed identical to those given in Tables S3-S4

Supplementary References

1 Kaya, M. & Higuchi, H. Nonlinear elasticity and an 8-nm working stroke of single myosin molecules in myofilaments. *Science* **329**, 686-689, doi:10.1126/science.1191484 (2010).

2 Llinas, P. *et al.* How actin initiates the motor activity of Myosin. *Dev Cell* **33**, 401-412, doi:10.1016/j.devcel.2015.03.025 (2015).

3 Smart, O. S., Goodfellow, J. M. & Wallace, B. A. The pore dimensions of gramicidin A. *Biophys. J.* **65**, 2455-2460, doi:10.1016/S0006-3495(93)81293-1 (1993).

4 Smart, O. S., Neduvelil, J. G., Wang, X., Wallace, B. A. & Sansom, M. S. HOLE: a program for the analysis of the pore dimensions of ion channel structural models. *J. Mol. Graph.* **14**, 354-360, 376, doi:10.1016/s0263-7855(97)00009-x (1996).

5 Humphrey, W., Dalke, A. & Schulten, K. VMD: visual molecular dynamics. *J. Mol. Graph.* **14**, 33-38, 27-38, doi:10.1016/0263-7855(96)00018-5 (1996).

6 Baker, N. A., Sept, D., Joseph, S., Holst, M. J. & McCammon, J. A. Electrostatics of nanosystems: application to microtubules and the ribosome. *Proc. Natl. Acad. Sci. U. S. A.* **98**, 10037-10041, doi:10.1073/pnas.181342398 (2001).

7 Usaj, M., Moretto, L., Vemula, V., Salhotra, A. & Mansson, A. Single molecule turnover of fluorescent ATP by myosin and actomyosin unveil elusive enzymatic mechanisms. *Commun Biol* **4**, 64, doi:10.1038/s42003-020-01574-0 (2021).

8 Muretta, J. M., Rohde, J. A., Johnsrud, D. O., Cornea, S. & Thomas, D. D. Direct real-time detection of the structural and biochemical events in the myosin power stroke. *Proc. Natl. Acad. Sci. U. S. A.* **112**, 14272-14277, doi:10.1073/pnas.1514859112 (2015).

9 White, H. D., Belknap, B. & Webb, M. R. Kinetics of nucleoside triphosphate cleavage and phosphate release steps by associated rabbit skeletal actomyosin, measured using a novel fluorescent probe for phosphate. *Biochemistry.* **36**, 11828-11836, doi:10.1021/bi970540h (1997).

10 Gargey, A., Iragavarapu, S. B., Grdzelishvili, A. V. & Nesmelov, Y. E. Electrostatic interactions in the SH1-SH2 helix of human cardiac myosin modulate the time of strong actomyosin binding. *J. Muscle Res. Cell Motil.* **42**, 137-147, doi:10.1007/s10974-020-09588-1 (2021).

11 Månsson, A. The effects of inorganic phosphate on muscle force development and energetics: challenges in modelling related to experimental uncertainties. *J. Muscle Res. Cell Motil.*, doi:10.1007/s10974-019-09558-2 (2019).

12 Månsson, A. Comparing models with one versus multiple myosin-binding sites per actin target zone: The power of simplicity. *J. Gen. Physiol.* **151**, 578-592, doi:10.1085/jgp.201812301 (2019).

13 Månsson, A. Actomyosin based contraction: one mechanokinetic model from single molecules to muscle? *J. Muscle Res. Cell Motil.* **37**, 181-194, doi:10.1007/s10974-016-9458-0 (2016).

14 Caremani, M., Melli, L., Dolfi, M., Lombardi, V. & Linari, M. The working stroke of the myosin II motor in muscle is not tightly coupled to release of orthophosphate from its active site. *J Physiol* **591**, 5187-5205, doi:10.1113/jphysiol.2013.257410 (2013).

15 Cooke, R., Franks, K., Luciani, G. B. & Pate, E. The inhibition of rabbit skeletal muscle contraction by hydrogen ions and phosphate. *J. Physiol. (Lond).* **395**, 77-97 (1988).

16 Coupland, M. E., Puchert, E. & Ranatunga, K. W. Temperature dependence of active tension in mammalian (rabbit psoas) muscle fibres: effect of inorganic phosphate. *J Physiol* **536**, 879-891 (2001).

17 Woody, M. S., Winkelmann, D. A., Capitanio, M., Ostap, E. M. & Goldman, Y. E. Single molecule mechanics resolves the earliest events in force generation by cardiac myosin. *Elife* **8**, doi:10.7554/eLife.49266 (2019).
18 Rahman, M. A., Usaj, M., Rassier, D. E. & Månsson, A. Blebbistatin Effects Expose Hidden Secrets in the Force-Generating Cycle of Actin and Myosin. *Biophys. J.* **115**, 386-397, doi:10.1016/j.bpj.2018.05.037 (2018).
19 Månsson, A., Morner, J. & Edman, K. A. Effects of amrinone on twitch, tetanus and shortening kinetics in mammalian skeletal muscle. *Acta Physiol. Scand.* **136**, 37-45 (1989).
20 Ranatunga, K. W. The force-velocity relation of rat fast- and slow-twitch muscles examined at different temperatures. *J. Physiol. (Lond).* **351**, 517-529 (1984).
21 Asmussen, G., Beckers-Bleukx, G. & Marechal, G. The force-velocity relation of the rabbit inferior oblique muscle; influence of temperature. *Pflugers Archiv : European journal of physiology* **426**, 542-547 (1994).
22 Homsher, E., Nili, M., Chen, I. Y. & Tobacman, L. S. Regulatory proteins alter nucleotide binding to acto-myosin of sliding filaments in motility assays. *Biophys. J.* **85**, 1046-1052 (2003).
23 Persson, M., Bengtsson, E., ten Siethoff, L. & Månsson, A. Nonlinear cross-bridge elasticity and post-power-stroke events in fast skeletal muscle actomyosin. *Biophys. J.* **105**, 1871-1881, doi:10.1016/j.bpj.2013.08.044 (2013).
24 Linari, M. *et al.* Straightening Out the Elasticity of Myosin Cross-Bridges. *Biophys. J.* **118**, 994-1002, doi:10.1016/j.bpj.2020.01.002 (2020).
25 Amyot, R. & Flechsig, H. BioAFMviewer: An interactive interface for simulated AFM scanning of biomolecular structures and dynamics. *PLoS computational biology* **16**, e1008444, doi:10.1371/journal.pcbi.1008444 (2020).
26 Månsson, A., Persson, M., Shalabi, N. & Rassier, D. E. Non-linear actomyosin elasticity in muscle? *Biophys. J.* **116**, 330–346 (2019).
