# Supplementary material for: Multistep orthophosphate release tunes actomyosin energy transduction
Source: Nat Commun. 2022 Aug 5;13:4575. doi: 10.1038/s41467-022-32110-9 (PMC9356070; doi:10.1038/s41467-022-32110-9)
Supplement: Supplementary file 3 — Description of Additional Supplementary Files [file 41467_2022_32110_MOESM3_ESM.pdf]

Description of Additional Supplementary Information

Legends for Supplementary Movies

Supplementary Movie 1

Single molecule Alexa647-ATP binding events under increasing added [Pi] (in mM).

Time laps TIRF microscopy image sequences of Alexa647- ATP bindings were combined in ImageJ. Each video shows the 50 first seconds of the 15 min videos underlying the data presented in Fig. 2a. The videos were accelerated ~5 times (from 19 fps to 100 fps) for practical reasons.

Supplementary Movie 2

Representative hs-AFM movie of the skeletal muscle HMM molecules with two heads simultaneously bound to parallel actin filaments in the presence of 2 µM Mg.ADP. Scan area of 150 x 75 nm2 173 with 80 x 40 pixels. The scale bar is 30 nm. Recording and playing rates: 6.7 fps. Original hs-AFM movie (a) with interactive 3D surface plot imageJ plugin (b) applied to highlight myosin heads, lever arm and parts of subfragment 2 (S2) domain. The angle of the lever arm relative to actin filaments and the barbed (+)-end of actin filaments is shown in selected cases in (c). The lower panel demonstrates an atomic model of actin-myosin S1-ADP complex (PDB 6C1H). Movie is most informative when run frame by frame.

Supplementary Movie 3

Representative hs-AFM movie of the skeletal HMM muscle molecules with two heads simultaneously bound to parallel actin filaments in the presence of 10 µM Mg.ADP. Scan area of 150 x 75 nm2 183 with 80 x 40 pixels. The scale bar is 30 nm. Recording rate: 3.3 fps, playing rate: 6.7 fps. Original hs-AFM movie (a) with interactive 3D surface plot imageJ plugin (b) applied to highlight myosin heads and lever arm S2 domain. The angle of the lever arm relative to actin filaments and the barbed (+)-end of actin filaments are shown in (c). The lower panel demonstrates an atomic model of actin-myosin S1-ADP complex (PDB 6C1H). The

movie is most informative when run frame by frame.

Supplementary Movie 4

Representative hs-AFM movie of the skeletal HMM molecules with two heads simultaneously bound to parallel actin filaments in the presence of 2 µM Mg.ADP and 100 µM meta-vanadate. Scan area of 150 x 75 nm2 with 80 x 40 pixels. The scale bar is 30 nm. Recording and playing rate: 6.7 fps. Original hs-AFM movie (a) with interactive 3D surface plot imageJ plugin (b) applied to highlight myosin heads and lever arm S2 domain. The angle of the lever arm relative to actin filaments and the barbed (+)-end of actin filaments are shown in (c). The lower panel demonstrates an atomic model of actin-myosin S1-ADP-V04 complex (PDB 1QVI). The movie is most informative when run frame by frame.

Supplementary Movie 5

Representative hs-AFM movie of the skeletal HMM molecules with two heads simultaneously bound to parallel actin filaments in the presence of 2 µM Mg.ADP and 100 µM meta-vanadate. Scan area of 150 x 120 nm2 with 80 x 64 pixels. The scale bar is 30 nm. Recording and playing rate: 6.7 fps. Original hs-AFM movie (a) with interactive 3D surface plot imageJ plugin (b) applied to highlight myosin heads and lever arm S2 domain. The angle of the lever arm relative to actin filaments and the barbed (+)-end of actin filaments are shown in (c). The lower panel demonstrates an atomic model of actin-myosin S1-ADP-V04 complex (PDB 1QVI). The movie is most informative when run frame by frame.

Supplementary Movie 6

Representative hs-AFM movie of the skeletal HMM molecules with two heads simultaneously bound to parallel actin filaments in the presence of 10 µM Mg.ADP and 10 µM para-aminoblebbistatin. Scan area of 79 x 63 nm2 with 80 x 32 pixels. The scale bar is 20 nm. Recording and playing rates: 10 fps. Original hs-AFM movie (a) with interactive 3D surface plot imageJ plugin (b) applied to highlight myosin heads and lever arm S2 domain. The angle of the lever arm relative to actin filaments and the barbed (+)-end of actin filaments are shown in (c). The lower panel demonstrates an atomic model of actin-myosin S1-ADP219 Blebbistatin (BS) complex (PDB 6Z7U) with the pre-power stroke lever arm (PDB 1QVI). The

movie is most informative when run frame by frame.

Supplementary Movie 7

Representative hs-AFM movie of the skeletal HMM molecules with two heads simultaneously bound to parallel actin filaments in the presence of 10 µM Mg.ADP and 10 µM para-aminoblebbistatin. Scan area of 150 x 120 nm2 225 with 80 x 64 pixels. The scale bar is 30 nm. Recording and playing rates: 6.7 fps. Original hs-AFM movie (a) with interactive 3D surface plot imageJ plugin (b) applied to highlight myosin heads and lever arm S2 domain. The angle of the lever arm relative to actin filaments and the barbed (+)-end of actin filaments are shown in (c). The lower panel demonstrates an atomic model of actin-myosin S1-ADP230 Blebbistatin (BS) complex (PDB 6Z7U) with the pre-power stroke lever arm (PDB 1QVI). The movie is most informative when run frame by frame.

Supplementary Movie 8

hs-AFM movies of the skeletal HMM molecules with two heads simultaneously bound to parallel actin filaments in the presence of 1 µM Mg.ADP (3rd movie) or 2 µM Mg.ADP (1st 238 and 2nd movies). Scan area of 200 x 200 nm2 239 with 120 x 120 pixels. The scale bars are 30 nm. Recording and playing rates: 2 fps.

Supplementary Movie 9

hs-AFM movies of the skeletal HMM molecules with two heads simultaneously bound to parallel actin filaments in the presence of 1 µM Mg.ADP imaged at 2 fps for a scan area of 200 x 200 nm2

with 120 x 120 pixels (1st 246 movie) and switched to 6.7 fps for a scan area of 150 x 75 nm2 with 80 x 40 pixels (2nd 247 movie). The scale bars are 30 nm.

Supplementary Movie 10

hs-AFM movies of the skeletal HMM molecules with two heads simultaneously bound to parallel actin filaments (1st and 2nd 251 movies) or HMM molecules bound to the one actin filament (3rd movie) in the presence of 10 µM Mg.ADP. Scan area of 150 x 75 nm2 252 with 80

253 x 40 pixels. The scale bars are 30 nm. Recording and playing rates: 3.3 fps.

Supplementary Movie 11

hs-AFM movies of the skeletal HMM molecules with two heads simultaneously bound to parallel actin filaments in the presence of 1 µM Mg.ADP (4th movie), 2 µM Mg.ADP (1st 258 and 3rd movies), 10 µM Mg.ADP (2nd movie) or in the nucleotide-free state (5th 259 movie). Scan area of 150 x 75 nm2 with 80 x 40 pixels (1st-4th movies) or 200 x 120 nm2 260 with 80 x 48 pixels (5th 261 movie). The scale bars are 30 nm. Recording and playing rates: 6.7 fps.

Supplementary Movie 12

hs-AFM movies of the skeletal HMM molecules bound to one actin filaments in the presence of 2 µM Mg.ADP. Scan area of 150 x 75 nm2 265 with 80 x 40 pixels. The scale bars are 30 nm. Recording and playing rates: 6.7 fps.

Supplementary Movie 13

hs-AFM movies of the skeletal HMM molecules with two heads simultaneously bound to parallel actin filaments in the presence of 2 µM Mg.ADP and 100 µM meta-vanadate. Scan area of 200 x 200 nm2 271 with 120 x 120 pixels. The scale bars are 30 nm. Recording and playing rates: 2 fps.

Supplementary Movie 14

hs-AFM movies of the skeletal HMM molecules with two heads simultaneously bound to parallel actin filaments in the presence of 2 µM Mg.ADP and 100 µM meta-vanadate imaged at 2 fps. Scan area of 200 x 200 nm2 with 120 x 120 pixels (1st 277 movie) and switched to 6.7 fps for a scan area of 150 x 75 nm2 with 80 x 40 pixels (2nd 278 movie). The scale bars are 30 nm.

Supplementary Movie 15

hs-AFM movies of the skeletal HMM molecules with two heads simultaneously bound to parallel actin filaments in the presence of 2 µM Mg.ADP and 100 µM meta-vanadate. Scan

area of 150 x 120 nm2 with 80 x 64 pixels. The scale bars are 30 nm. Recording and playing rate: 6.7 fps.

Supplementary Movie 16

hs-AFM movies of the skeletal HMM molecules bound to one actin filaments in the presence of 2 µM Mg.ADP and 100 µM meta-vanadate. Scan area of 150 x 120 nm2 with 80 x 64 pixels. The scale bars are 30 nm. Recording and playing rate: 6.7 fps.

Supplementary Movie 17

hs-AFM movies of the skeletal HMM molecules with two heads simultaneously bound to parallel actin filaments in the presence of 10 µM Mg.ADP and and 10 µM paraaminoblebbistatin. Scan area of 150 x 120 nm2 with 80 x 64 pixels (1st and 2nd movies) and for a scan area of 79 x 63 nm2 with 80 x 32 pixels (3rd movie). The scale bars are 30 nm (1st and 2nd movies) and 20 nm (3rd movie). Recording rate: 6.7 fps, playing rate: 3.3 fps
