# Supplementary material for: Multistep orthophosphate release tunes actomyosin energy transduction
Source: Nat Commun. 2022 Aug 5;13:4575. doi: 10.1038/s41467-022-32110-9 (PMC9356070; doi:10.1038/s41467-022-32110-9)
Supplement: Supplementary file 21 — Reporting summary [file 41467_2022_32110_MOESM21_ESM.pdf]

nature research

Corresponding author(s): Alf Mansson, R Friedman, DE Rassier

Last updated by author(s): Jul 6, 2022

Reporting Summary

Nature Research wishes to improve the reproducibility of the work that we publish. This form provides structure for consistency and transparency in reporting. For further information on Nature Research policies, see our Editorial Policies and the Editorial Policy Checklist.

Statistics

For all statistical analyses, confirm that the following items are present in the figure legend, table legend, main text, or Methods section.

n/a	Confirmed	
☐	☒	The exact sample size (n) for each experimental group/condition, given as a discrete number and unit of measurement
☐	☒	A statement on whether measurements were taken from distinct samples or whether the same sample was measured repeatedly
☐	☒	The statistical test(s) used AND whether they are one- or two-sided<br>*Only common tests should be described solely by name; describe more complex techniques in the Methods section.*
☒	☐	A description of all covariates tested
☐	☒	A description of any assumptions or corrections, such as tests of normality and adjustment for multiple comparisons
☐	☒	A full description of the statistical parameters including central tendency (e.g. means) or other basic estimates (e.g. regression coefficient) AND variation (e.g. standard deviation) or associated estimates of uncertainty (e.g. confidence intervals)
☐	☒	For null hypothesis testing, the test statistic (e.g. F, t, r) with confidence intervals, effect sizes, degrees of freedom and P value noted<br>*Give P values as exact values whenever suitable.*
☒	☐	For Bayesian analysis, information on the choice of priors and Markov chain Monte Carlo settings
☒	☐	For hierarchical and complex designs, identification of the appropriate level for tests and full reporting of outcomes
☒	☐	Estimates of effect sizes (e.g. Cohen's d, Pearson's r), indicating how they were calculated

Our web collection on statistics for biologists contains articles on many of the points above.

Software and code

Policy information about availability of computer code

Data collection	Single molecule fluorescence (TIRF): NIS Elements (Nikon, ver. 4.51)<br>hs-AFM (RIBM, Japan), IgorPro software (Wave Metrics, v. 6.3.7.2)

April 2020

Data analysis	Molecular modelling: PyMOL (The PyMOL Molecular Graphics System, Version 1.8.6.2 and 2.0 Schrödinger, LLC.; APBS extension: Jurrus et al. PROTEIN SCIENCE 2018 27:112—128, web server: https://server.poissonboltzmann.org/; PDB2PQR extension: Dolinsky et al. Nucleic Acid Res 2004 (DOI: 10.1093/nar/gkh381), web server: https://server.poissonboltzmann.org/ PDB (online server). CHARMM (ver. 45b1), CHARMM-GUI (on-line server), HOLE (v. 2.2.005;on-line server http://www.holeprogram.org/ Smart et al. Biophys J. 1993. 65:2455-60), SWISS-MODEL (on-line server; https://swissmodel.expasy.org/), UCSF-Chimera (v.1.14), VMD (v. 1.9.3) and for the calculations of the binding energy, NWCHEM (ver. 6.8.1), The molecular modelling software is available in useful versions, free on-line.<br><br>Single molecule fluorescence: Matlab with toolboxes and scripts (ver. 2020a, 2021a), Fiji (ImageJ; ver. 1.53i), MS Excel 2016, Graphpad Prism (v. 8 and 9). The code of the Matlab routines used for single molecule analysis will be made available upon reasonable request.<br><br>Mechanokinetic modelling: Ordinary differential equations were solved using the program Simnon v.3.00.001. Simnon was marketed by SSPA martime consulting, Gothenburg, Sweden and originally developed by the Department of Automatic Control, Lund University, Lund, Sweden. It is no longer commercially available. The code for mechanokinetic modelling is provided in the Supplementary Materials of the paper.<br><br>hs-AFM: To remove spike noise in the images and to make the xy-plane flat, the hs-AFM images were processed with a low-pass filtering by custom-made Kodec software (4.4.7.39). The x,y coordinates to calculate the center of mass analyses of hs-AFM images were made using pixel-search Kodec software available from here: https://doi.org/10.7554/eLife.04806.031. Histograms, time course data and other statistical analyses were performed using GraphPad Prism software (v.9.2).

For manuscripts utilizing custom algorithms or software that are central to the research but not yet described in published literature, software must be made available to editors and reviewers. We strongly encourage code deposition in a community repository (e.g. GitHub). See the Nature Research guidelines for submitting code & software for further information.

Data

Policy information about availability of data

All manuscripts must include a data availability statement. This statement should provide the following information, where applicable:

- Accession codes, unique identifiers, or web links for publicly available datasets
- A list of figures that have associated raw data
- A description of any restrictions on data availability

Data from modeling are available in the main paper (Figures 1-2, 4-5), Excel source data files for Figures 4-5 and the Supporting information (Supplementary Figures 1-11; Supplementary Tables 1-5). Raw data (movies) from single molecule fluorescence experiments are provided upon reasonable request. The high-speed AFM data (raw data and processed data) are included in Supplementary Material (Supplementary Figures 12-16, Supplementary Movies 2-17 and the Excel source data file for hs-AFM data). Throughout the manuscript we have used deposited myosin structures with the following accession codes: 5N6A [http://doi.org/10.2210/pdb5N6A/pdb] (Pre-powerstroke); 4PFO [http://doi.org/10.2210/pdb4PFO/pdb] (Pi release state); 3I5F [http://doi.org/10.2210/pdb3I5F/pdb] ; 1QVI [http://doi.org/10.2210/pdb1QVI/pdb] (Pre-powerstroke); 6Z7U [http://doi.org/10.2210/pdb6Z7U/pdb] (with Blebbistatin).

Field-specific reporting

Please select the one below that is the best fit for your research. If you are not sure, read the appropriate sections before making your selection.

☒ Life sciences ☐ Behavioural & social sciences ☐ Ecological, evolutionary & environmental sciences

For a reference copy of the document with all sections, see nature.com/documents/nr-reporting-summary-flat.pdf

Life sciences study design

All studies must disclose on these points even when the disclosure is negative.

Sample size	Single molecule fluroescence: No sample size calculation was performed prior to the experiments as the appropriate sample size for this type of experiments has been determined previously (Usaj et al, Commun Biol, 4:64, 2021)<br>hs-AFM: No statistical methods were used to predetermine the sample size. This is justified because parameter values are not estimated by small random sample statistics but rather by fitting Gaussian or double-Gaussian probability density functions to large data sets with experimental data.<br>Molecular modelling and Mechanokinetic modelling: The sample size and other statistical issues and issues related to experimental designs are not relevant in these cases because no stochastic, e.g. Monte-Carlo simulations were performed. That is the results of the modeling are fully deterministic, without variability between runs.
Data exclusions	Single molecule fluorescence: Data were only excluded based on the strictly applied criteria as described in the Methods, that each individual trace representing one myosin molecule must contain at least 10 independent events per 15 min trace.<br><br>hs-AFM: There were no data sets exclusions. Only a few images in the successive hs-AFM sets were omitted from data analysis due to their blurriness or unclearness.
Replication	Single molecule fluorescence: Each binding event of a fluorescent ATP molecule to a myosin molecule was assumed to be an independent random event independent of the experimental occasion, or myosin batch. The duration of these events were then used to produce probability distributions that were analyzed to give the quantitative data based on exponential fits to the distributions. The exponential model has strong theoretical foundations in this regard. Except for the cases with the highest [Pi], The number of events used for construction of the distributions ranged between about 100 and 1000 with limited effects of the sample size on the quantitative characteristics of the distributions. This finding supports our assumption that all individual events are independent. Support for that assumption also derives from

previous findings of very similar kinetic properties of myosin from different preparations (Persson et al., Biophysical Journal, 2013, 105:1871-81; Rahman et al., J Muscle Research Cell Motility, 2018, 39:175–187). Furthermore, the assumption is supported by similar results in experiments performed on different days using different myosin preparations (in this and other work; Usaj et al, Commun Biol, 4:64, 2021). Finally, in support of our assumed definition of independent events, no consistent changes in behavior were seen during the course of a given experiment .
hs-AFM: The experiments were replicated multiple times as indicated. We assumed each image frame of an actin attached myosin molecule as one independent sample from a single distribution of myosin head conformations in equilibrium, unaffected by experimental occasion and/or myosin preparation per se but only by the experimental condition (e.g. ADP alone, ADP+PAB etc.). This is justified by several lines of argumentation. First, myosin heads are believed (see paper for references) to be independent force-generators, meaning that the possible attachment of one head does not affect the conformation of another head (cf. Supplementary Figure 16a). Second, we found similar results in three hs-AFM experiments performed on different days using different surface preparations, solutions and HMM fragments from two different myosin preparations. This is consistent with Supplementary Figures 16 and 18 as well as previous results showing appreciable similarity between different experimental occasions and different myosin preparations (for reference see paper) as well as negligible changes in function over prolonged experimental periods (references in paper) Third, and finally, the ergodicity principle allows distributions of myosin conformations (e.g. as shown in Figure 6 and Supplementary Figures 16 and 18) to be obtained either from a time series or an ensemble of myosin conformations or a combination as used here. The quantitative parameter estimates were then obtained by fitting Gaussian functions or double-Gaussian functions to these distributions (based on > 200 data points) rather than using small sample based estimates of mean values and errors.

Randomization

Single molecule fluorescence: Our study does not include sample allocations in different groups. When we were collecting microscopy data from sample (imaging chamber), fields of view were chosen randomly. After such choice all events were analyzed provided that they met predefined criteria (see above under data exclusions)
hs-AFM: The study does not include sample allocations in different groups and randomization is of no relevance.

Blinding

ingle molecule fluorescence: The experiments were performed by skilled persons. Blinding is not relevant due to nature of the experiments, extra time and cost which would need to train additional PhD students, postdocs and researchers. Thus most of the experiments were performed by a single person which collect and analyzed the data. However the traces were extracted by one person while dwell time collections were mostly performed by another person without knowing the experimental conditions behind the traces.
hs-AFM: Performed experiments were not blinded for reasons similar to those given for the single molecule fluorescence experiments.

Reporting for specific materials, systems and methods

We require information from authors about some types of materials, experimental systems and methods used in many studies. Here, indicate whether each material, system or method listed is relevant to your study. If you are not sure if a list item applies to your research, read the appropriate section before selecting a response.

Materials & experimental systems

n/a	Involved in the study
☒	☐ Antibodies
☒	☐ Eukaryotic cell lines
☒	☐ Palaeontology and archaeology
☐	☒ Animals and other organisms
☒	☐ Human research participants
☒	☐ Clinical data
☒	☐ Dual use research of concern

Methods

n/a	Involved in the study
☒	☐ ChIP-seq
☒	☐ Flow cytometry
☒	☐ MRI-based neuroimaging

Animals and other organisms

Policy information about studies involving animals; ARRIVE guidelines recommended for reporting animal research

Laboratory animals

Single molecule fluorescence experiments: New Zealand white rabbits (female, 2 kg, 8-9 weeks).
hs-AFM experiments: New Zealand white rabbits (female, 2.6-2.7 kg, 78-84 days).

Wild animals

The study did not involve wild animals.

Field-collected samples

The study did not involve field-collected samples.

Ethics oversight

Single molecule fluorescence experiments: Regional Ethical Committee for Animal experiments in Linköping, Sweden, reference number 73-14.
hs-AFM experiments: McGill University, reference number MCGL-5227

Note that full information on the approval of the study protocol must also be provided in the manuscript.
